# Ypsiyunnosides A–E, Five New Cholestanol Glycosides from *Ypsilandrayunnanensis*

**DOI:** 10.1007/s13659-016-0098-2

**Published:** 2016-04-20

**Authors:** Yu Chen, Yong-Ai Si, Wei Ni, Huan Yan, Xu-Jie Qin, Chang-Xiang Chen, Hai-Yang Liu

**Affiliations:** 1 State Key Laboratory of Phytochemistry and Plant Resources in West China, Kunming Institute of Botany, Chinese Academy of Sciences, Kunming, 650201 China; 2 University of Chinese Academy of Sciences, Beijing, 100039 China

**Keywords:** *Ypsilandra yunnanensis*, Liliaceae, Cholestane glycoside, Ypsiyunnosides A–E

## Abstract

**Abstract:**

Phytochemical investigation on the whole plants of *Ypsilandra yunnanensis* was carried out for the first time and led to the isolation of five new cholestanol glycosides, ypsiyunnosides A–E (**1**–**5**), and one known analogue. Their structures were determined mainly by detailed spectroscopic analysis, including extensive 1D and 2D NMR, MS and UV, as well as chemical methods. Among them, compound **1** possessed a rare 6/6/6/5/5 fused-rings cholestanol sketelon, which was identified as (23*R*,25*R*)-3*β*,16*α*,26-triol-16,23-cyclocholest-5,17(20)-dien-22-one. Their induced platelet aggregation activities and cytotoxicities were evaluated.

**Graphical Abstract:**

Five new cholestanol glycosides, ypsiyunnosides A–E (**1**–**5**), were isolated from the whole plants of *Ypsilandra* *yunnanensis*. Compound **1** possessed a rare 6/6/6/5/5 fused-rings cholestanol sketelon. Their structures were elucidated by a combination of 1D and 2D NMR, MS and chemical analysis.
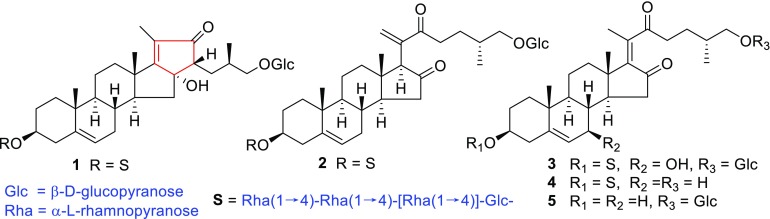

**Electronic supplementary material:**

The online version of this article (doi:10.1007/s13659-016-0098-2) contains supplementary material, which is available to authorized users.

## Introduction

**Fig. 1:**
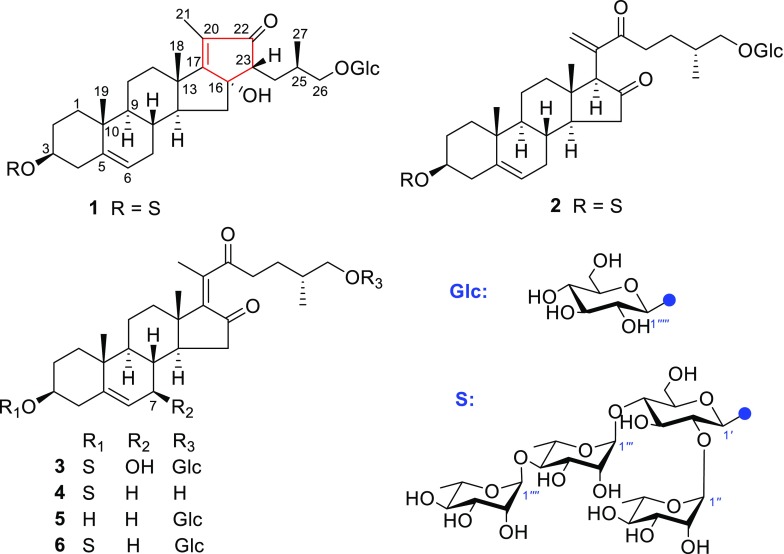
Chemical structures of compounds **1**–**5**

*Ypsilandra yunnanensis* W. W. Sm. et J. F. Jeffr., belonging to the family of Liliaceae, is an erect, perennial herb and distributed mainly in the northwest of Yunnan Province and the southeast of the Tibet Autonomous Region of China. It usually grows in grass slope, forest-shrub edge, or under the bushes at the altitude between 3300 and 4000 m [1]. Previously, we have performed a systemic phytochemical investigation of the whole plants of *Y. thibetica* and obtained a series of spirostanol-, furostanol-, and 23-spirocholestanol glycosides with cytotoxic, antifungal, hemostatic, and anti-HIV activities [2–9]. However, the phytochemicals and the biological activity of *Y. yunnanensis* have not been reported so far. The HPLC analysis revealed that the secondary metabolites of *Y. thibetica* and *Y. yunnanensis* were very different, especially in the water-soluble part of the crude extract. As part of our continuing search for structurally diverse and bioactive steroidal glycosides from the *Ypsilandra* plants, the chemical constituents of *Y. yunnanensis* were investigated. The result led to the isolation of five new cholestane glycosides (**1–5**) and one known analogue, parispseudoside C (**6**) [10] as shown in Fig. 1 from the 70 % EtOH extract of the whole title plant. Their structures were determined by the analysis of spectroscopic data (HR-ESI-MS, 1D and 2D NMR) and chemical methods. This paper presents herein the isolation, structural elucidation, and the induced platelet aggregation activities and cytotoxicities of these compounds.

## Results and Discussion

Ypsiyunnoside A (**1**) was obtained as a white amorphous powder, $\left[ \alpha \right]_{\text{D}}^{23}$ – 139.6 (*c* 0.05, MeOH); UV (MeOH) *λ*_max_ (log *ε*) 237 (3.03) and 197 (2.76) nm. Its molecular formula was determined as C_57_H_90_O_26_ on the basis of a positive-ion at *m/z* 1213.5616 [M + Na]^+^ (calcd. 1213.5618) in its HR-ESI-MS and ^13^C NMR data (Table 2), corresponding to 13° of unsaturation. The IR spectrum of **1** exhibited absorption bands for hydroxyl (3440 cm^−1^) and *α*,*β*-unsaturated ketone (1701 and 1658 cm^−1^) functionalities, which was confirmed by the UV absorption at *λ*_max_ (MeOH) 237 nm. The ^1^H NMR spectrum of the aglycone moiety of **1** (Table 1) displayed signals for four characteristic steroidal methyls at *δ*_H_ 1.08 (s, Me-19), 1.46 (s, Me-18), 1.83 (s, Me-21), and 1.12 (d, *J* = 6.5 Hz, Me-27) and an olefinic proton at *δ*_H_ 5.35 (br s). The ^13^C NMR and DEPT spectra (Table 2) showed a total of 57 carbon signals, which were classified as 7 methyls, 11 methylenes, 32 methines, and 7 quaternary carbons. Among them, 27 carbon signals were assigned to the aglycone including four methyls at *δ*_C_ 8.6 (q, Me-21), 15.7 (q, Me-18), 17.1 (q, Me-27) and 19.5 (q, Me-19), three oxygenated carbons at *δ*_C_ 76.6 (t, C-26), 78.1 (d, C-3) and 83.0 (s, C-16), and four olefinic carbons at *δ*_C_ 121.7 (d, C-6), 128.3 (s, C-20), 141.1 (s, C-5) and 182.1 (s, C-17), as well as one carbonyl carbon at *δ*_C_ 212.3 (s, C-22). Furthermore, the ^1^H NMR and ^13^C NMR spectra of **1** exhibited five anomeric protons at *δ*_H_ 4.84 (d, *J* = 7.7 Hz, H-1*′′′′′*), 4.97 (d, *J* = 7.4 Hz, H-1*′*), 5.85 (br s, H-1*′′′*), 6.30 (br s, H-1*′′′′*) and 6.41 (br s, H-1*′′*) (Table 1), corresponding to five carbons at *δ*_C_ 105.3 (d, C-1*′′′′′*), 100.5 (d, C-1*′*), 102.4 (d, C-1*′′′*), 103.3 (d, C-1*′′′′*) and 102.2 (d, C-1*′′*) (Table 2), respectively. Acid hydrolysis of **1** gave d-glucose and l-rhamnose, which were determined by GC chromatographic analysis of their l-cysteine methyl ester-TMS derivates. The above spectroscopic information hinted that compound **1** was a cholestanol pentaglycoside.

**Table 1:** ^1^H NMR data of compounds **1–5** in C_5_D_5_N (*δ* in ppm and *J* in Hz)

Position	**1** ^a^	**2** ^b^	**3** ^b^	**4** ^b^	**5** ^b^
1*a*	1.77 (o)	1.72 (d, 11.5)	1.74 (o)	1.73 (o)	1.79 (m)
1*b*	1.03 (m)	0.97 (o)	0.98 (o)	0.97 (o)	1.09 (m)
2*a*	2.08 (t, 8.6)	2.07 (o)	2.09 (o)	2.08 (o)	1.78 (m)
2*b*	1.89 (o)	1.85 (o)	1.89 (o)	1.88 (o)	1.51 (m)
3	3.92 (m)	3.90 (m)	3.94 (m)	3.88 (m)	3.94 (m)
4*a*	2.80 (m)	2.83 (m)	2.88 (o)	2.90 (o)	2.79 (m)
4*b*	2.74 (m)	2.75 (m)	2.80 (o)	2.76 (o)	2.78 (m)
6	5.35 (br s)	5.31 (br s)	5.69 (br s)	5.29 (br d, 4.8)	5.35 (br d, 4.5)
7*a*	1.95 (o)	1.83 (o)	4.04 (m)	1.75 (o)	2.08 (o)
7*b*	1.58 (o)	1.56 (o)		1.46 (o)	1.88 (m)
8	1.68 (m)	1.50 (o)	1.77 (o)	1.48 (o)	1.52 (o)
9	0.98 (m)	1.04 (o)	1.19 (m)	0.98 (o)	1.03 (o)
11*a*	1.56 (m)	1.48 (o)	1.57 (o)	1.56 (o)	1.57 (o)
11*b*	1.08 (m)	1.37 (m)	1.48 (o)	1.48 (o)	1.47 (o)
12*a*	2.08 (m)	1.54 (o)	2.15 (o)	2.15 (o)	2.15 (o)
12*b*	1.45 (m)	1.45 (o)	1.55 (o)	2.02 (o)	1.98 (o)
14	1.08 (d, 7.8)	1.48 (o)	1.63 (d, 6.0)	1.32 (t, 7.0)	1.38 (s)
15*a*	2.28 (dd, 10.0, 8.8)	2.35 (dd, 16.8, 6.8)	2.92 (o)	2.10 (o)	2.14 (o)
15*b*	1.98 (o)	2.01 (m)	2.70 (m)	1.74 (o)	1.53 (o)
17		3.71 (br s)			
18	1.46 (s)	0.66 (s)	0.96 (s)	0.95 (s)	0.95 (s)
19	1.08 (s)	1.05 (s)	1.05 (s)	1.07 (s)	1.02 (s)
21a	1.83 (s)	6.56 (s)	2.01 (s)	1.99 (s)	1.99 (s)
21*b*		5.90 (d, 4.2)			
23*a*	2.44 (o)	2.90 (o)	2.90 (o)	2.91 (o)	2.61 (o)
23*b*		2.88 (o)	2.82 (o)	2.72 (o)	2.61 (o)
24*a*	2.39 (m)	1.97 (o)	2.17 (o)	2.33 (m)	2.14 (o)
24*b*	1.98 (m)	1.64 (o)	1.88 (o)	1.94 (o)	1.86 (o)
25	2.51 (m)	1.95 (o)	2.03 (o)	1.97 (o)	2.00 (o)
26*a*	4.03 (m)	3.99 (m)	3.97 (m)	3.80 (o)	3.95 (m)
26*b*	3.72 (dd, 7.8, 6.2)	3.56 (dd, 9.5, 6.0)	3.60 (dd, 9.4, 6.1)	3.75 (dd, 9.4, 6.1)	3.60 (t, 7.2)
27	1.12 (d, 6.5)	0.96 (d, 6.6)	0.94 (d, 6.0)	1.11 (d, 6.5)	0.98 (d, 6.7)
3-*O*-Glc					
1*′*	4.97 (d, 7.4)	4.98 (d, 7.5)	4.99 (d, 7.4)	5.00 (d, 7.2)	
2*′*	4.22 (o)	4.27 (o)	4.28 (o)	4.26 (o)	
3*′*	4.22 (o)	4.27 (o)	4.28 (o)	4.24 (o)	
4*′*	4.42 (m)	4.47 (m)	4.47 (o)	4.47 (o)	
5*′*	3.62 (br d, 9.2)	3.64 (dt, 9.8, 2.5)	3.63 (dt, 9.8, 2.7)	3.64 (dt, 9.8, 2.5)	
6*′*	4.19 (m)	4.22 (m)	4.21 (o)	4.23 (m)	
	4.07 (m)	4.08 (o)	4.10 (o)	4.08 (m)	
2*′*-*O*-Rha					
1*′′*	6.41 (br s)	6.46 (br s)	6.45 (br s)	6.47 (br s)	
2*′′*	4.89 (m)	4.91 (br d, 3.5)	4.90 (br d, 2.5)	4.92 (br s)	
3*′′*	4.66 (dd, 9.6, 3.8)	4.70 (dd, 9.6, 3.8)	4.67 (dd, 9.6, 3.8)	4.71 (br s)	
4*′′*	4.39 (o)	4.42 (o)	4.42(o)	4.43 (m)	
5*′′*	4.97 (o)	4.99 (o)	4.99 (m)	4.97 (m)	
6*′′*	1.78 (d, 6.2)	1.80 (d, 6.2)	1.80 (d, 6.2)	1.81 (d, 6.2)	
4*′*-*O*-Rha					
1*′′′*	5.85 (br s)	5.90 (br s)	5.91 (br s)	5.91 (br s)	
2*′′′*	4.56 (m)	4.62 (m)	4.60 (br s)	4.60 (br s)	
3*′′′*	4.57 (m)	4.60 (brs)	4.60 (br s)	4.60 (br s)	
4*′′′*	4.45 (m)	4.50 (o)	4.50 (o)	4.51 (o)	
5*′′′*	4.95 (o)	5.01 (o)	5.01 (o)	5.01 (o)	
6*′′′*	1.59 (d, 5.7)	1.62 (d, 6.1)	1.63 (d, 6.1)	1.63 (d, 6.0)	
4*′′′*-*O*-Rha					
1*′′′′*	6.30 (br s)	6.34 (br s)	6.35 (br s)	6.35 (br s)	
2*′′′′*	4.89 (br s)	4.96 (br d, 1.8)	4.96 (br s)	4.97 (m)	
3*′′′′*	4.51 (dd, 9.6, 3.8)	4.55 (dd, 9.6, 3.8)	4.35 (dd, 9.6, 3.8)	4.57 (dd, 9.6,3.8)	
4*′′′′*	4.31 (o)	4.35 (q, 9.5)	4.36 (q, 9.4)	4.36 (q, 9.4)	
5*′′′′*	4.35 (o)	4.40 (o)	4.41 (o)	4.41 (m)	
6*′′′′*	1.59 (d, 5.7)	1.62 (d, 6.1)	1.63 (d, 6.1)	1.63 (d, 6.0)	
26-*O*-Glc					
1*′′′′′*	4.84 (d, 7.7)	4.86 (d, 7.9)	4.86 (d, 7.8)		4.82 (d, 7.8)
2*′′′′′*	4.04 (o)	4.08 (o)	4.07 (o)		4.03 (m)
3*′′′′′*	4.24 (o)	4.28 (o)	4.27 (o)		4.24 (o)
4*′′′′′*	4.22 (m)	4.27 (m)	4.28 (o)		4.23 (o)
5*′′′′′*	3.89 (o)	3.99 (o)	3.99 (m)		3.94 (m)
6*′′′′′*	4.53 (o)	4.60 (o)	4.61 (o)		4.55 (dd, 11.7, 2.2)
	4.35 (m)	4.43 (m)	4.43 (o)		4.39 (dd, 11.7, 5.2)

^a^Recorded on 400 MHz

^b^Recorded on 600 MHz.; *o* overlap

**Table 2:** ^13^C NMR data of compounds **1–6** in C_5_D_5_N (*δ* in ppm)

Position	**1** ^a^	**2** ^b^	**3** ^b^	**4** ^b^	**5** ^b^	**6** ^b^
1	37.6 (t)	37.6 (t)	37.4 (t)	37.6 (t)	37.3 (t)	37.2 (t)
2	30.2 (t)	30.5 (t)	30.6 (t)	30.6 (t)	31.6 (t)	30.1 (t)
3	78.1 (d)	78.4 (d)	78.1 (d)	78.3 (d)	70.9 (d)	77.9 (d)
4	39.1 (t)	39.4 (t)	39.2 (t)	39.3 (t)	38.6 (t)	38.9 (t)
5	141.1 (s)	141.4 (s)	142.0 (s)	141.3 (s)	141.9 (s)	140.9 (s)
6	121.7 (d)	121.9 (d)	128.9 (d)	121.9 (d)	120.5 (d)	121.4 (d)
7	31.9 (t)	32.6 (t)	73.2 (d)	32.2 (t)	32.4 (t)	31.7 (t)
8	32.0 (d)	31.9 (d)	40.0 (d)	31.2 (d)	30.7 (d)	30.8 (d)
9	50.6 (d)	50.8 (d)	48.6 (d)	50.3 (d)	50.4 (d)	50.5 (d)
10	37.3 (s)	37.6 (s)	37.5 (s)	37.5 (s)	36.8 (s)	37.1 (s)
11	20.7 (t)	21.3 (t)	21.4 (t)	21.3 (t)	20.8 (t)	20.9 (t)
12	35.8 (t)	38.3 (t)	36.4 (t)	38.4 (t)	38.6 (t)	38.8 (t)
13	44.1 (s)	43.6 (s)	44.2 (s)	43.8 (s)	43.3 (s)	43.4 (s)
14	53.9 (d)	50.6 (d)	50.6 (d)	50.9 (d)	49.8 (d)	49.9 (d)
15	38.5 (t)	39.7 (t)	38.9 (t)	36.1 (t)	35.9 (t)	36.1 (t)
16	83.0 (s)	215.5 (s)	211.2 (s)	211.2 (s)	210.4 (s)	210.5 (s)
17	182.1 (s)	63.5 (d)	142.6 (s)	143.0 (s)	142.4 (s)	142.6 (s)
18	15.7 (q)	14.6 (q)	17.1 (q)	17.2 (q)	15.6 (q)	16.8 (q)
19	19.5 (q)	19.8 (q)	19.4 (q)	19.8 (q)	19.4 (q)	19.4 (q)
20	128.3 (s)	143.2 (s)	146.1 (s)	146.1 (s)	145.6 (s)	145.7 (s)
21	8.6 (q)	129.7 (t)	16.3 (q)	16.3 (q)	16.7 (q)	15.8 (q)
22	212.3 (s)	202.0 (s)	207.4 (s)	206.2 (s)	205.6 (s)	205.8 (s)
23	57.6 (d)	35.6(t)	41.6 (t)	39.5 (t)	43.3 (t)	38.0 (t)
24	29.3 (t)	29.6 (t)	28.4 (t)	28.3 (t)	27.8 (t)	27.9 (t)
25	32.1 (d)	34.0 (d)	33.9 (d)	36.6 (d)	33.3 (d)	33.4 (d)
26	76.6 (t)	75.4 (t)	75.6 (t)	67.9 (t)	74.9 (t)	75.1 (t)
27	17.1 (q)	17.8 (q)	17.9 (q)	17.8 (q)	17.3 (q)	17.5 (q)
3-*O*-Glc						
1*′*	100.5 (d)	100.8 (d)	100.8 (d)	100.8 (d)		100.4 (d)
2*′*	78.5 (d)	78.4 (d)	78.4 (d)	78.3 (d)		77.9 (d)
3*′*	78.2 (d)	78.2 (d)	78.1 (d)	78.2 (d)		77.8(d)
4*′*	77.8 (d)	78.0 (d)	77.9 (d)	77.9 (d)		77.7 (d)
5*′*	77.0 (d)	77.6 (d)	77.5 (d)	77.5 (d)		77.1 (d)
6*′*	61.4 (t)	61.6 (t)	61.6 (t)	61.6 (t)		61.3 (t)
2*′*-*O*-Rha						
1*′′*	102.2 (d)	102.7 (d)	102.7 (d)	102.7 (d)		102.2 (d)
2*′′*	72.6 (d)	73.0 (d)	73.0 (d)	73.0 (d)		72.6 (d)
3*′′*	72.9 (d)	73.4(d)	73.4 (d)	73.4 (d)		72.9 (d)
4*′′*	74.1 (d)	74.6 (d)	74.5 (d)	74.6 (d)		74.0 (d)
5*′′*	69.6 (d)	70.0 (d)	70.0 (d)	70.1 (d)		69.6 (d)
6*′′*	18.7 (q)	19.1 (q)	19.2 (q)	19.2 (q)		18.7 (q)
4*′*-*O*-Rha						
1*′′′*	102.4 (d)	102.7 (d)	102.8 (d)	102.7 (d)		102.2 (d)
2*′′′*	72.9 (d)	73.4 (d)	73.4 (d)	73.4 (d)		72.9 (d)
3*′′′*	73.3 (d)	73.9 (d)	73.8 (d)	73.8(d)		73.0 (d)
4*′′′*	80.5 (d)	80.9 (d)	80.9 (d)	80.9 (d)		80.5 (d)
5*′′′*	68.5 (d)	68.8 (d)	68.8 (d)	68.8 (d)		68.4 (d)
6*′′′*	18.9 (q)	18.9 (q)	19.3 (q)	19.7 (q)		18.9 (q)
4*′′′*-*O*-Rha						
1*′′′′*	103.3 (d)	103.9 (d)	103.9 (d)	103.9 (d)		103.4 (d)
2*′′′′*	72.7 (d)	73.2(d)	73.2 (d)	73.2 (d)		72.7 (d)
3*′′′′*	72.9 (d)	73.4 (d)	73.4 (d)	73.4 (d)		72.9 (d)
4*′′′′*	74.2 (d)	74.5 (d)	74.6 (d)	74.5 (d)		73.4 (d)
5*′′′′*	70.4 (d)	70.9 (d)	70.9 (d)	70.9 (d)		70.5 (d)
6*′′′′*	18.5 (q)	19.4 (q)	18.9 (q)	18.9 (q)		18.5 (q)
26-*O*-Glc						
1*′′′′′*	105.3 (d)	105.4 (d)	105.4 (d)		104.7 (d)	104.9 (d)
2*′′′′′*	75.3 (d)	75.7 (d)	75.7 (d)		75.1 (d)	75.3 (d)
3*′′′′′*	78.7 (d)	79.1 (d)	79.1 (d)		78.4 (d)	78.6 (d)
4*′′′′′*	71.9 (d)	72.1 (d)	72.1 (d)		71.6 (d)	70.9 (d)
5*′′′′′*	78.7 (d)	79.2 (d)	79.0 (d)		78.5 (d)	78.7 (d)
6*′′′′′*	63.0 (t)	63.3 (t)	63.2 (t)		62.7 (t)	62.8 (t)

^a^Recorded on 100 MHz

^b^Recorded on 150 MHz

**Fig. 2:**
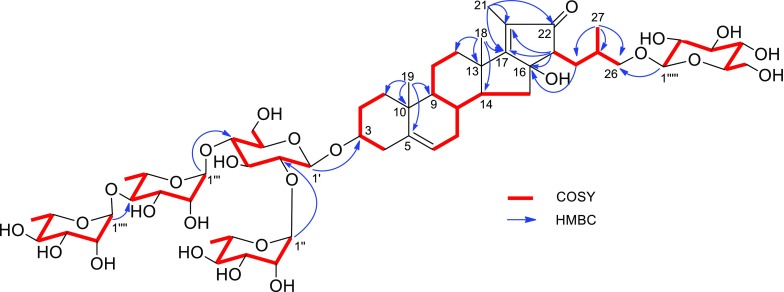
The ^I^H–^1^H COSY correlations and selected key HMBC correlations of **1**

**Fig. 3:**
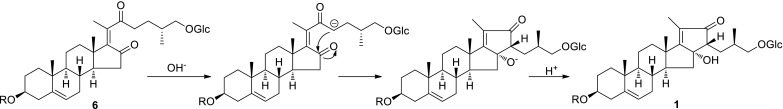
Possible biosynthetic pathway for the aglycone of **1**

**Fig. 4:**
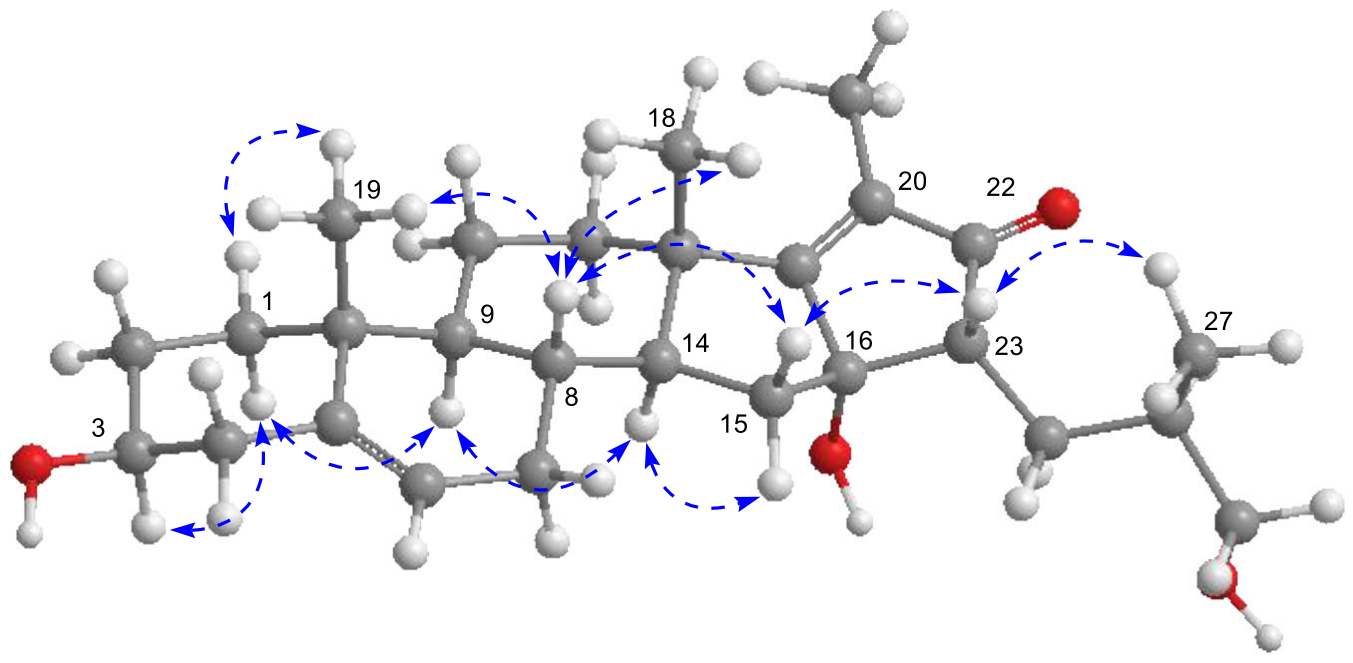
Key ROESY correlations for the aglycone of **1**

Comparison of the ^1^H and ^13^C NMR spectra of the aglycone of **1** with those of parispseudoside C (**6**) [10] led to find the absence of one carbonyl group, one methylene and the appearance of an oxygen-bearing quaternary carbon (*δ*_C_ 83.0) and one methine (*δ*_H_ 2.44; *δ*_C_ 57.6) in **1**. In addition, one carbonyl group, two double bonds and five monosaccharides in compound **1** accounted for 8 degrees of unsaturation, and the remaining five degrees of unsaturation required that the aglycone of **1** be pentacyclic. Therefore, it was supposed that compound **1** was a 23,16-aldol condensation product of parispseudoside C (**6**), which was verified by the ^1^H–^1^H COSY and HMBC spectra. The ^1^H–^1^H COSY correlations (Fig. 2) of *δ*_H_ 4.03 (H_a_-26) and 3.72 (H_b_-26) with *δ*_H_ 2.51 (H-25), of *δ*_H_ 2.51 (H-25) with *δ*_H_ 2.39 (H_a_-24) and 1.98 (H_b_-24), of *δ*_H_ 2.51 (H-25) with *δ*_H_ 1.12 (Me-27), and of *δ*_H_ 1.98 (H_b_-24) with *δ*_H_ 2.44 (H-23), together with the HMBC correlation (Fig. 2) of *δ*_H_ 2.51 (H-25) with *δ*_C_ 57.6 (C-23) indicated that the methine was assigned to C-23. The observed HMBC correlations (Fig. 2) from Me-21 to C-17, C-20, and C-22, from H-23 to C-16, and C-22, from H_2_-24 to C-16 and C-22 supported that the formation of a cyclopentane with *α*,*β*-unsaturated ketone group by 23,16-aldol condensation (Fig. 3). The ROESY correlations (Fig. 4) of Me-18/H-8, H-8/H_*β*_-15 (*δ*_H_ 1.98), and H_*β*_-15/H-23 suggested that H-23 was *β*-oriented. Furthermore, the obvious correlation from H_*β*_-15 to H-23 hinted that OH-16 was *α*-oriented. The 25*R*-configuration of **1** was deduced from the geminal proton resonances of H_a_-26 and H_b_-26, which showed Δab (*δ*_Ha_ – *δ*_Hb_) of 0.31, less than 0.48 [11].

The *β*-configurations for the two glucopyranosyls were deduced by large *J*_1H-2H_ values (^3^ *J*_1,2_ = 7.4–7.7 Hz) of their anomeric protons, while the anomeric configuration of the three rhamnopyranosyls were assigned as *α*-configuration from the ^13^C NMR data of C-3*′′*, C-5*′′*, C-3*′′′*, C-5*′′′*, C-3*′′′′*, and C-5*′′′′* with those of the corresponding carbons of methyl *α*- and *β*-rhamnopyranoside [12, 13]. The sequence of the sugars and the linkage sites to the aglycone were in good agreement with those of **6**, which was supported by their almost identical NMR data and the HMBC correlations from H-1′ (*δ*_H_ 4.97) of 3-Glu to C-3 (*δ*_C_ 78.1) of aglycone, from H-1″ (*δ*_H_ 6.41) of 2′-Rha to C-2′ (*δ*_C_ 78.5) of 3-Glu, from H-1′′′ (*δ*_H_ 5.85) of 4′-Rha to C-4′ (*δ*_C_ 77.8) of 3-Glc, from H-1′′′′ (*δ*_H_ 6.30) of 4′′′ -Rha to C-4′′′ (*δ*_C_ 80.5) of 4′-Rha, and from H-1′′′′′ (*δ*_H_ 4.84) of 26-Glu to C-26 (*δ*_C_ 76.6) of aglycone. Thus, the structure of ypsiyunnoside A (**1**) was elucidated as 26-*O*-*β*-d-glucopyranosyl-(23*R*,25*R*)-3*β*,16*α*,26-triol-16,23-cyclocholest-5,17(20)-dien-22-one-3-*O*-*α*-l-rhamnopyranosyl-(1 → 4)-*α*-l-rhamnopyranosyl-(1 → 4)-[*α*-l-rhamnopyranosyl-(1 → 2)]-*β*-d-glucopyranoside.

Ypsiyunnoside B (**2**) was isolated as a white amorphous powder, $\left[ \alpha \right]_{\text{D}}^{24}$ – 148.7 (*c* 0.05, MeOH); UV (MeOH) *λ*_max_ (log *ε*) 217 (2.78) and 203 (2.92) nm. It had a molecular formula of C_57_H_90_O_26_ as deduced by HR-ESI-MS ion peak at *m/z* 1213.5616 [M + Na]^+^ (calcd. 1213.5618) and ^13^C NMR data (Table 2). A careful comparison of the NMR and MS data of **2** (Tables 1 and 2) with those of **6** revealed that they were similar, except for the presence of a methine (*δ*_H_ 3.71; *δ*_C_ 63.5) and a terminal double bond [*δ*_H_ 5.90 and 6.56 (each 1H); *δ*_C_ 129.7 (t) and *δ*_C_ 143.2 (s)] and the absence of a methyl and a Δ^17(20)^ double bond in **2**. These spectroscopic characteristics suggested that the aglycone of **2** was (25*R*)-3*β*,26-diol-cholest-5,20-dien-16,22-dione. The HMBC correlations of Me-18 with C-17 (*δ*_C_ 63.5), of H-17 with C-12, C-13, C-14, C-16, C-18, C-20, C-21, and C-22, and of H_2_-21 (*δ*_H_ 5.90 and 6.56) with C-17 (*δ*_C_ 63.5) and C-22 (*δ*_C_ 202.0) evidenced this suggestion. According to the consistency of the ^1^H and ^13^C NMR data (Tables 1 and 2) of the sugar moieties in **1**, **2**, and **6**, these three compounds were ascertained to have the same sugar sequence and linkage position. Consequently, the structure of ypsiyunnoside B (**2**) was determined to be 26-*O*-*β*-d-glucopyranosyl-(25*R*)-3*β*,26-diol-cholest-5,20-dien-16,22-dione-3-*O*-*α*-l-rhamnopyranosyl-(1 → 4)-*α*-l-rhamnopyranosyl-(1 → 4)-[*α*-l-rhamnopyranosyl-(1 → 2)]-*β*-d-glucopyranoside.

Ypsiyunnoside C (**3**) was isolated as white amorphous powder, $\left[ \alpha \right]_{\text{D}}^{23}$ – 109.2 (*c* 0.05, MeOH); UV (MeOH) *λ*_max_ (log *ε*) 245 (2.69) and 204 (2.85) nm. Its molecular formula was established as C_57_H_90_O_27_ by the positive HR-ESI-MS at *m/z* 1229.5563 [M + Na]^+^ (calcd. 1229.5567) and ^13^C NMR data (Table 2). Inspection of the ^1^H and ^13^C NMR spectroscopic data of **3** (Tables 1 and 2) with those of **6** revealed their considerable structural similarity. The major differences were observed for the replacement of a methene in **6** by an oxygenated methine (*δ*_H_ 4.04; *δ*_C_ 73.2). The oxymethine was placed at C-7 on the basis of the ^1^H–^1^H COSY correlation of *δ*_H_ 5.69 (H-6) with *δ*_H_ 4.04 (H-7). The relative configuration of OH-7 was determined to be *β*-oriented by the chemical shift of C-7 (*δ*_C_ 73.2), while the signals for C-7 would be at *δ*_C_ 64.7 of the 7α-isomer [7]. Other parts were identical to those of **6** based on 2D NMR experiments. Therefore, the structure of ypsiyunnoside C (**3**) was characterized as 26-*O*-*β*-d-glucopyranosyl-(25*R*)-3*β*,7*β*,26-triol-cholest-5,17(20)-dien-16,22-dione-3-*O*-*α*-l-rhamnopyranosyl-(1 → 4)-*α*-l-rhamnopyranosyl-(1 → 4)-[*α*-l-rhamnopyranosyl-(1 → 2)]-*β*-d-glucopyranoside.

Ypsiyunnoside D (**4**), obtained as white amorphous powder, gave the molecular formula of C_51_H_80_O_21_ from its HR-ESI-MS ion peak at *m/z* 1051.5083 [M + Na]^+^ (calcd. 1051.5090) and ^13^C NMR data (Table 2). The ^1^H and ^13^C NMR spectroscopic data of **4** (Tables 1 and 2) were similar to those of **6**, differing only in the disappearance of glucopyranosyl signals and the upfield shift of C-26 (*δ*_C_ 75.1 ppm → 67.9 ppm). This indicated that no sugar moiety was attached to C-26. By the detailed analysis of 1D and 2D-NMR data (Tables 1 and 2) of the sugar moieties in compounds **4** and **6**, the two cholestanol glycosides were considered to have the same tetraglycoside and the linkage position at C-3. On the basis of the above evidence, the structure of ypsiyunnoside D (**4**) was elucidated as (25*R*)-3*β*,26-diol-cholest-5,17(20)-dien-16,22-dione-3-*O*-*α*-l-rhamnopyranosyl-(1 → 4)-*α*-l-rhamnopyranosyl-(1 → 4)-[*α*-l-rhamnopyranosyl-(1 → 2)]-*β*-d-glucopyranoside.

Ypsiyunnoside E (**5**) was obtained as white amorphous powder with $\left[ \alpha \right]_{\text{D}}^{23}$ – 84.8 (*c* 0.05, MeOH) and UV (MeOH) *λ*_max_ (log *ε*) 242 (2.78) and 203 (2.88) nm. It had a molecular formula of C_33_H_50_O_9_ based on the positive-ion HR-ESI-MS (*m/z* 613.3357 [M + Na]^+^; calcd. 613.3353) and ^13^C NMR data (Table 2). The ^1^H and ^13^C NMR spectroscopic data of **5** showed its aglycone resembling that of compounds **4** and **6**. However, the ^1^H NMR spectrum (Table 1) showed only one anomeric proton signal at *δ*_H_ 4.82 (d, *J* = 7.8 Hz), which was identified as a *β*-d-glucopyranosyl by the aforementioned method. The HMBC correlation of H-1*′* (*δ*_H_ 4.82) of the glucopyranosyl with C-26 (*δ*_C_ 74.9) revealed that the glucopyranosyl was linked to C-26. In conclusion, the structure of ypsiyunnoside E (**5**) was established as 26-*O*-*β*-d-glucopyranosyl-(25*R*)-3*β*,26-diol-cholest-5,17(20)-dien-16,22-dione.

Considering the traditional use of *Ypsilandra* plants as hemostatic medicine for Yi minority [14] and the cytotoxic activity of steroidal glycosides previously obtained from *Y.* *thibetica* [3, 6], compounds **1**–**6** were evaluated for their induced platelet aggregation activities and cytotoxicities against HEK293 and HepG2 human cancer cell lines. Unfortunately, the results showed that none of them had any obviously induced platelet aggregation activity at the concentration of 300 μg/mL and cytotoxic activity against the two human cancer cell lines (HepG2 and HEK293, IC_50_ > 20 μM).

## Experimental

### Plant Material

The whole plants of *Y. yunnanensis* were collected in September 2012 from Gongshan county, Yunnan Province, China, and identified by Dr. Rong Li (Institute of Botany, Chinese Academy of Sciences). A voucher specimen (No. HY0019) has been deposited at the State Key Laboratory of Phytochemistry and Plant Resources in West China, Kunming Institute of Botany, Chinese Academy of Sciences.

### Extraction and Isolation

The air-dried and powdered whole plants of *Y. yunnanensis* (5.0 kg) were extracted three times with 70 % EtOH (20 L × 3) under reflux for a total of 6 h (3 × 2 h) and the combined extract was concentrated under reduced pressure to give an aqueous solution (4.3 L). The solution was partitioned with *n*-BuOH (5 L × 4). After evaporation of the *n*-BuOH soluble fraction, the total saponin (905 g) was obtained. The saponin was subjected to CC (silica gel, 200–300 mesh; gradient CHCl_3_-MeOH 10:1–1:1, v/v) to afford 19 fractions (Fr. 1–19). The water-soluble part (Fr. 18 and Fr.19) showed different peaks from the extract of *Y. thibetica* on the HPLC chromatograms. Thus, the two fractions were further purified by different separation methods. Fr. 18 (208 g) was separated by CC (silica gel, 200–300 mesh; gradient CHCl_3_-MeOH, 9:1 → 1:1 v/v) to give five subfractions. Fr. 18-1 was subjected to Sephadex LH-20 column (MeOH) and semi-prep. HPLC (MeCN-H_2_O, 10:90 → 25:75, 0–10 min; MeCN-H_2_O 25:75 → 35:65, 10–30 min) to yield **5** (5.6 mg). Fr. 18-4 was subjected to an RP-18 column (MeOH-H_2_O 10:90 → 40:60 v/v) and further purified by semi-prep. HPLC (MeCN-H_2_O 15:85 → 50:50, 35 min) to afford **3** (3.2 mg), **4** (3.8 mg), and **6** (2.5 mg). Fr. 19 (130 g) was subjected to a MCI gel CC and then RP-18 column (MeOH-H_2_O, 10:90 → 50:50 v/v) to give four fractions. Fr. 19-2 was repeatedly subjected to Sephadex LH-20 CC (MeOH) to give **2** (11.9 mg). Fr.19-3 was subjected to CC (silica gel, 200–300 mesh; gradient CHCl_3_-MeOH, 9:1 → 1:1 v/v) and followed by semi-prep. HPLC (MeCN-H_2_O 20:80 → 30:70, 30 min) to yield **1** (75 mg).

### Ypsiyunnoside A (**1**)

White amorphous powder, $\left[ \alpha \right]_{\text{D}}^{23}$ – 139.6 (*c* 0.05, MeOH); UV (MeOH) *λ*_max_ (log *ε*) 237 (3.03) and 197 (2.76) nm; IR (KBr) *ν*_max_ 3440, 2935, 1701, 1658, 1452, 1383, 1041, 984, 911, 884, 838 cm^−1^; ^1^H NMR data see Table 1; ^13^C NMR data see Table 2; ESIMS *m/z* 1213 [M + Na]^+^; HRESIMS *m/z* 1213.5616 (calcd for C_57_H_90_O_26_Na [M + Na]^+^, 1213.5618).

### Ypsiyunnoside B (**2**)

White amorphous powder, $\left[ \alpha \right]_{\text{D}}^{24}$ – 148.7 (*c* 0.05, MeOH); UV (MeOH) *λ*_max_ (log *ε*) 217 (2.78) and 203 (2.92) nm; IR(KBr) *ν*_max_ 3441, 2934, 1734, 1632, 1452, 1383, 1043, 985, 910, 882, 836 cm^−1^; ^1^H NMR data see Table 1; ^13^C NMR data see Table 2; ESIMS *m/z* 1213 [M + Na]^+^; HRESIMS *m/z* 1213.5616 (calcd for C_57_H_90_O_26_Na [M + Na]^+^, 1213.5618).

### Ypsiyunnoside C (**3**)

White amorphous powder, $\left[ \alpha \right]_{\text{D}}^{23}$ – 109.2 (*c* 0.05, MeOH); UV (MeOH) *λ*_max_ (log *ε*) 245 (2.69) and 204 (2.85) nm; IR(KBr) *ν*_max_ 3441, 2931, 1631, 1453, 1384, 1043, 984, 912, 882, 837 cm^−1^; ^1^H NMR data see Table 1; ^13^C NMR data see Table 2; ESIMS *m/z* 1229 [M + Na]^+^; HRESIMS *m/z* 1229.5563 (calcd for C_57_H_90_O_27_Na [M + Na]^+^, 1229.5567).

### Ypsiyunnoside D (**4**)

White amorphous powder with $\left[ \alpha \right]_{\text{D}}^{23}$ – 142.8 (*c* 0.05, MeOH); UV (MeOH) *λ*_max_ (log *ε*) 246 (2.78) and 202 (2.87) nm; IR(KBr) *ν*_max_ 3445, 2931, 1715, 1632, 1451, 1383, 1048, 984, 913, 880, 837 cm^−1^; ^1^H NMR data see Table 1; ^13^C NMR data see Table 2; ESIMS *m/z* 1027 [M – H]^–^; HRESIMS *m/z* 1051.5083 (calcd for C_51_H_80_O_21_Na [M + Na]^+^, 1051.5090).

### Ypsiyunnoside E (**5**)

White amorphous powder with $\left[ \alpha \right]_{\text{D}}^{23}$ – 84.8 (*c* 0.05, MeOH); UV (MeOH) *λ*_max_ (log *ε*) 242 (2.78) and 203 (2.88) nm; IR(KBr) *ν*_max_ 3445, 2928, 1709, 1634, 1515,1451, 1383, 1040, 922, 898, 877 cm^−1^; ^1^H NMR data see Table 1; ^13^C NMR data see Table 2; ESIMS *m/z* 613 [M + Na]^+^; HRESIMS *m/z* 613.3357 (calcd for C_33_H_50_O_9_Na [M + Na]^+^, 613.3353).

### Acid Hydrolysis and GC Analysis

Compounds **1–5** (1–2 mg, each) were refluxed with 2 M HCl (1:1 v/v, 2 mL) on water bath for 2 h. After cooling, the reaction mixture was neutralized with 1 M NaOH and filtered. The filtrate was extracted with CHCl_3_ (3 × 2 mL). The aqueous layer was evaporated to dryness. The dried residue was dissolved in 0.5 mL anhydrous pyridine and treated with l-cysteine methyl ester hydrochloride (1.0 mg) stirred at 60 °C for 1 h. Trimethylsilylimidazole (0.5 mL) was added to the reaction mixtures, and these were kept at 60 °C for 30 min. The supernatants (2 μL) were analyzed by GC, respectively, under the following conditions: H_2_ flame ionization detector. Column: 30QC2/AC-5 quartz capillary column (30 m × 0.32 mm). Column temperature: 180–280 °C with the rate of 3 °C/min, and the carrier gas was N_2_ (1 mL/min); injector temperature: 250 °C; split ratio: 1/50. The configurations of d-glucose and l-rhamnose for compounds **1**–**5** were determined by comparison of the retention times of the corresponding derivatives with those of standard d-glucose and l-rhamnose giving a single peak at 19.01 and 15.43 min, respectively. These assignments of absolute configurations are based on the assumption that the corresponding enantiomeric sugar derivatives of d-cysteinyl methyl ester would in fact be separable from the l-cysteinyl derivatives under our GC conditions.

### Platelet Aggregation Assays

Turbidometric measurements of platelet aggregation of the samples were performed in a Chronolog Model 700 Aggregometer (Chronolog Corporation, Havertown, PA, USA) according to Born’s method [15, 16]. The blood from the rabbits by cardiac puncture, was anticoagulated with 3.8 % sodium citrate (9:1, v/v). Platelet-rich plasma (PRP) was prepared shortly after blood collection by spinning the sample at 180 g for 10 min at 22 °C. The PRP was carefully removed and the remaining blood centrifuged at 2400 g for 10 min to obtain platelet-poor plasma (PPP). The centrifuge temperature was maintained at 22 °C. Platelet counts were adjusted by the addition of PPP to the PRP to achieve a count of 500 × 10^9^ L^−1^. Platelet aggregation studies were completed within 3 h of preparation of PRP. Immediately after preparation of PRP, 250 μL was incubated in each of the test tubes at 37 °C for 5 min and then 2.5 μL of inducers (or compounds) was added. The change of optical density as a result of platelet aggregation was recorded. The extent of aggregation was estimated by the percentage of maximum increase in light transmission, with the buffer representing 100 % transmittance. Arachidonic acid (AA) was used as a positive control.

### Cytotoxic Assay

Compounds **1**–**6** were evaluated for their cytotoxicities against two human cancer cell lines (HepG2 and HEK293) using the MTT method described in the literature elsewhere [17]. (−)-OddC (Troxacitabine) was used as a positive control with IC_50_ values of 0.17 ± 0.02 μM and 0.30 ± 0.03 μM to the two cell lines, respectively. The experiments were conducted in three independent replicates, and IC_50_ > 20 μM was considered to be inactive.

## Electronic supplementary material

Below is the link to the electronic supplementary material.

Supplementary material 1 (DOC 2190 kb)
