# Supplementary material for: Ypsiyunnosides A–E, Five New Cholestanol Glycosides from Ypsilandrayunnanensis
Source: Nat Prod Bioprospect. 2016 Apr 20;6(3):173–82. doi: 10.1007/s13659-016-0098-2 (PMC5385661; doi:10.1007/s13659-016-0098-2)
Supplement: Supplementary file 1 — Supplementary material 1 (DOC 2190 kb) [file 13659_2016_98_MOESM1_ESM.doc]

**Supplementary Data for**

**Ypsiyunnosides A-E, Five New** **Cholestanol Glycosides from** ***Ypsilandra* *yunnanensis***

Yu Chen ^a, b,1^, Yong-Ai Si ^a, b,1^, Wei Ni ^a^, Huan Yan ^a^, Xu-Jie Qin ^a^, Chang-Xiang Chen ^a^, Hai-Yang Liu *^, a^

^a^ State Key Laboratory of Phytochemistry and Plant Resources in West China, Kunming Institute of Botany, Chinese Academy of Sciences, Kunming 650201, China

^b^ University of Chinese Academy of Sciences, Beijing 100039, China

* Corresponding author.

Tel/Fax: +86-871-65223246.

E-mail address: [haiyangliu@mail.kib.ac.cn](mailto:haiyangliu@mail.kib.ac.cn) (H.-Y. Liu).

^1^ These authors contributed equally to this work.

**Table of contents**

**Figure 1S.** ^1^H NMR spectrum of compound **1** (pyridine-*d*_5_, 400 MHz).

**Figure 2S.** ^13^C NMR spectrum of compound **1** (pyridine-*d*_5_, 100 MHz).

**Figure 3S.** HSQC spectrum of compound **1** (pyridine-*d*_5_, 500 MHz).

**Figure 4S.** HMBC spectrum of compound **1** (pyridine-*d*_5_, 500 MHz).

**Figure 5S.** ^1^H-^1^H COSY spectrum of compound **1** (pyridine-*d*_5_, 500 MHz).

**Figure 6S.** ROESY spectrum of compound **1** (pyridine-*d*_5_, 500 MHz).

**Figure 7S.** ^1^H NMR spectrum of compound **2** (pyridine-*d*_5_, 600 MHz).

**Figure 8S.** ^13^C NMR spectrum of compound **2** (pyridine-*d*_5_, 150 MHz).

**Figure 9S.** HSQC spectrum of compound **2** (pyridine-*d*_5_, 600 MHz).

**Figure 10S.** HMBC spectrum of compound **2** (pyridine-*d*_5_, 600 MHz).

**Figure 11S.** ^1^H-^1^H COSY spectrum of compound **2** (pyridine-*d*_5_, 600 MHz).

**Figure 12S.** ^1^H NMR spectrum of compound **3** (pyridine-*d*_5_, 600 MHz).

**Figure 13S.** ^13^C NMR spectrum of compound **3** (pyridine-*d*_5_, 150 MHz).

**Figure 14S.** HSQC spectrum of compound **3** (pyridine-*d*_5_, 600 MHz).

**Figure 15S.** HMBC spectrum of compound **3** (pyridine-*d*_5_, 600 MHz).

**Figure 16S.** ^1^H-^1^H COSY spectrum of compound **3** (pyridine-*d*_5_, 600 MHz).

**Figure 17S.** ^1^H NMR spectrum of compound **4** (pyridine-*d*_5_, 600 MHz).

**Figure 18S.** ^13^C NMR spectrum of compound **4** (pyridine-*d*_5_, 150 MHz).

**Figure 19S.** HSQC spectrum of compound **4** (pyridine-*d*_5_, 600 MHz).

**Figure 20S.** HMBC spectrum of compound **4** (pyridine-*d*_5_, 600 MHz).

**Figure 21S.** ^1^H NMR spectrum of compound **5** (pyridine-*d*_5_, 600 MHz).

**Figure 22S.** ^13^C NMR spectrum of compound **5** (pyridine-*d*_5_, 150 MHz).

**Figure 23S.** HSQC spectrum of compound **5** (pyridine-*d*_5_, 600 MHz).

**Figure 24S.** HMBC spectrum of compound **5** (pyridine-*d*_5_, 600 MHz).


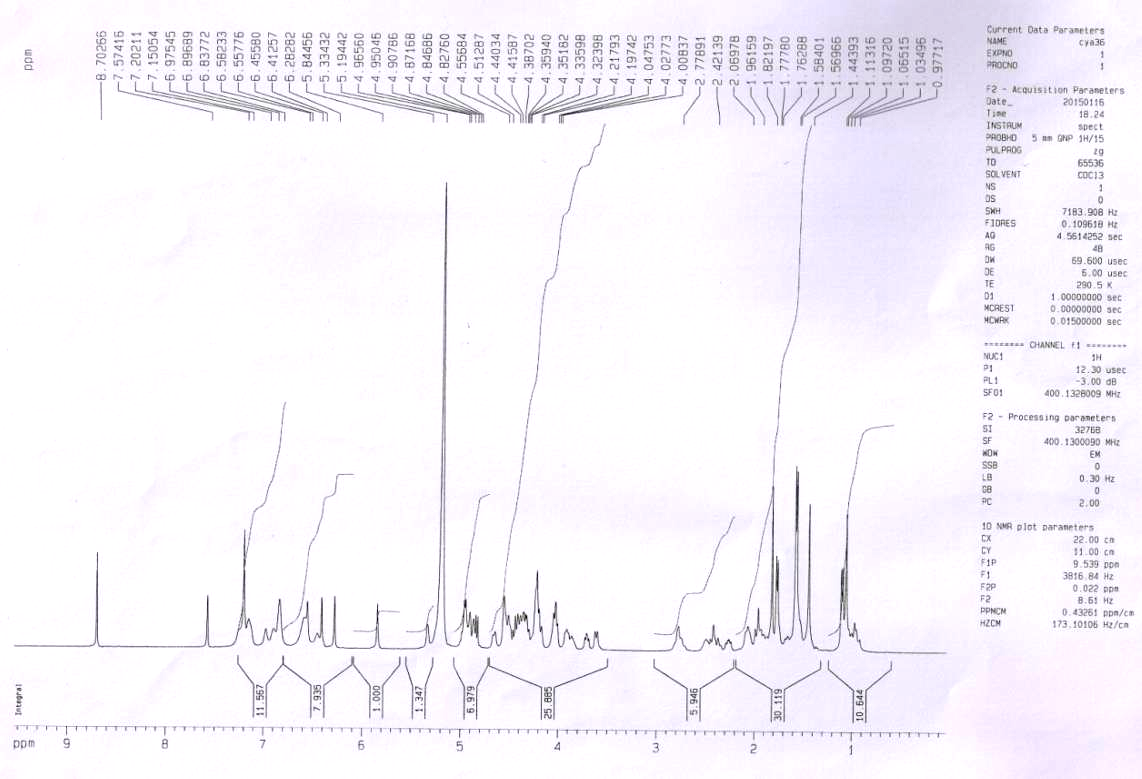


**Figure 1S.** ^1^H NMR spectrum of compound **1** (pyridine-*d*_5_, 400 MHz).


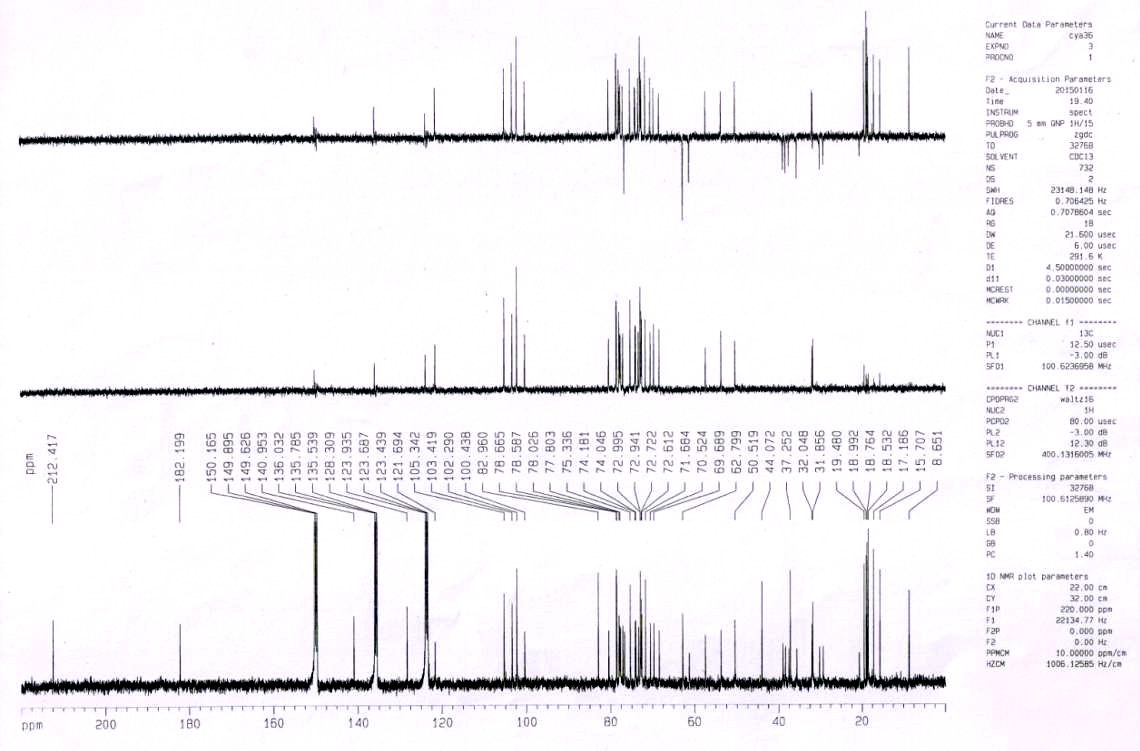


**Figure 2S.** ^13^C NMR spectrum of compound **1** (pyridine-*d*_5_, 100 MHz).


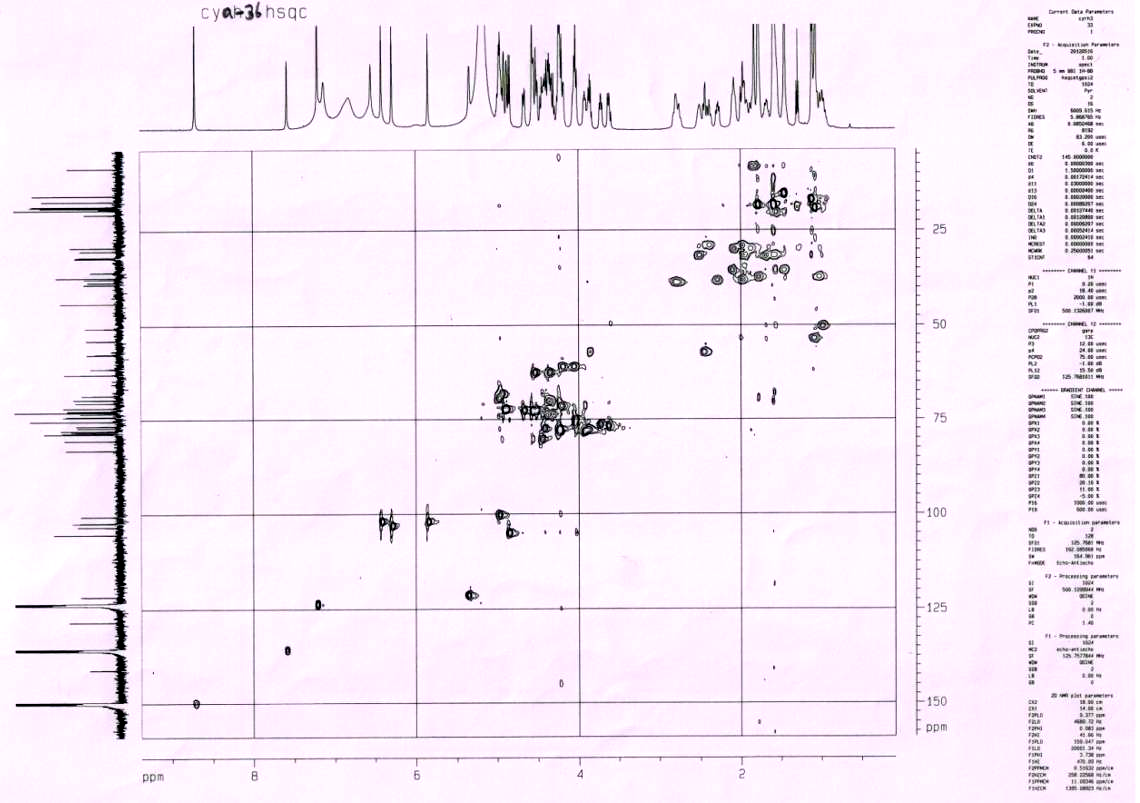


**Figure 3S.** HSQC spectrum of compound **1** (pyridine-*d*_5_, 500 MHz).


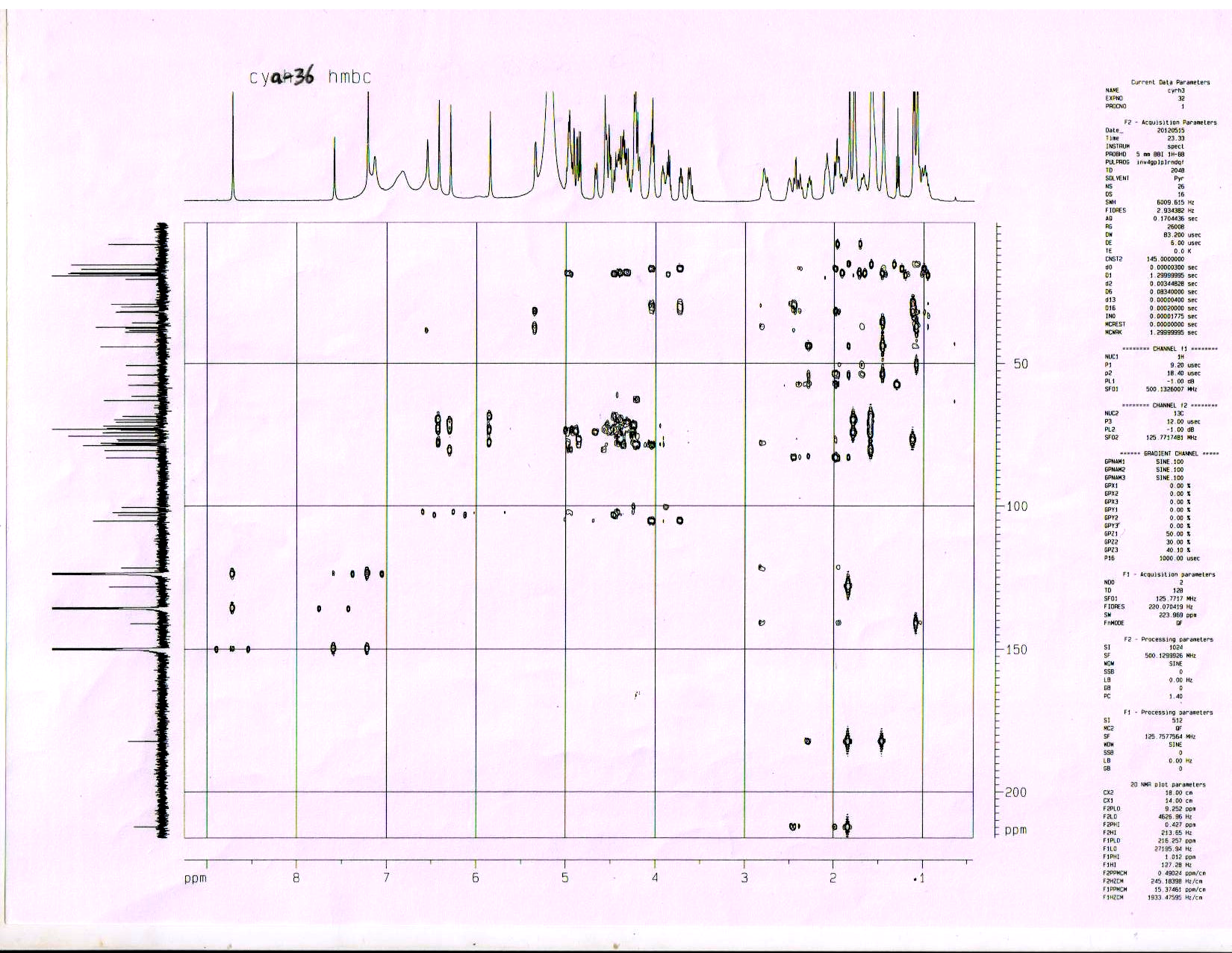


**Figure 4S.** HMBC spectrum of compound **1** (pyridine-*d*_5_, 500 MHz).


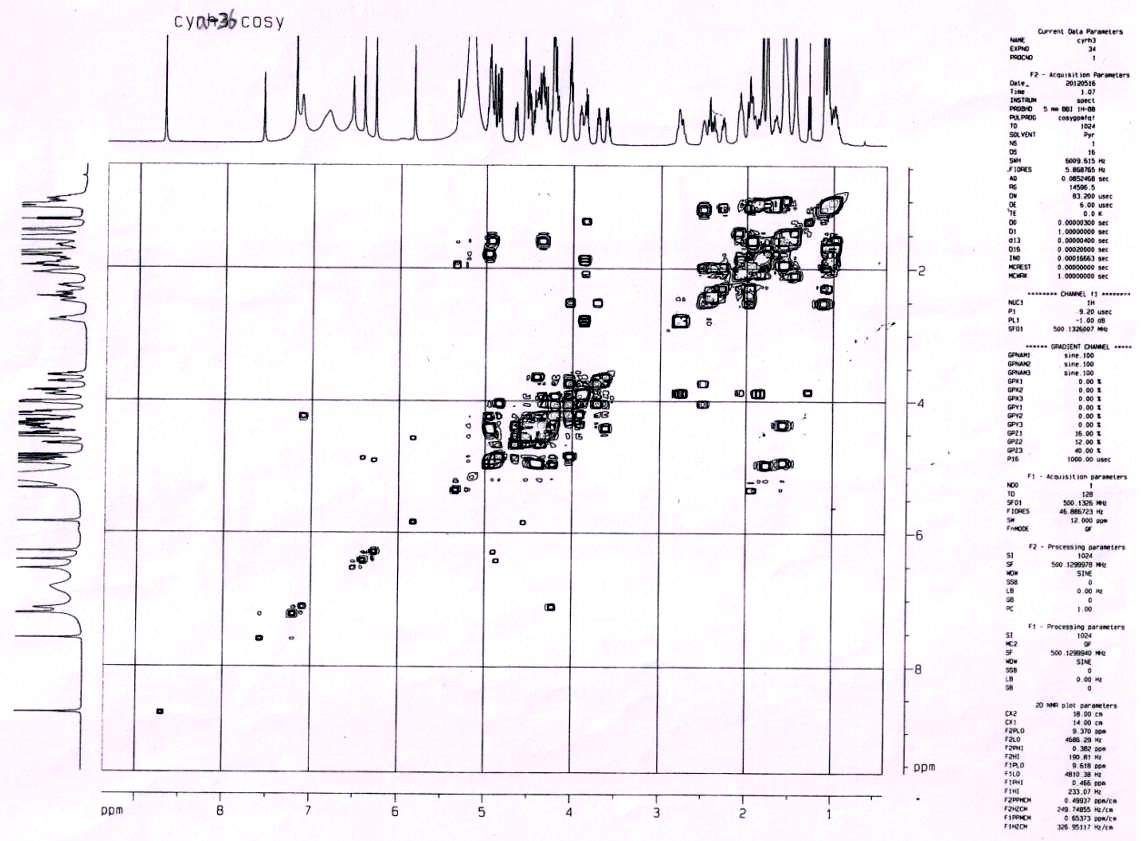


**Figure 5S.** ^1^H-^1^H COSY spectrum of compound **1** (pyridine-*d*_5_, 500 MHz).


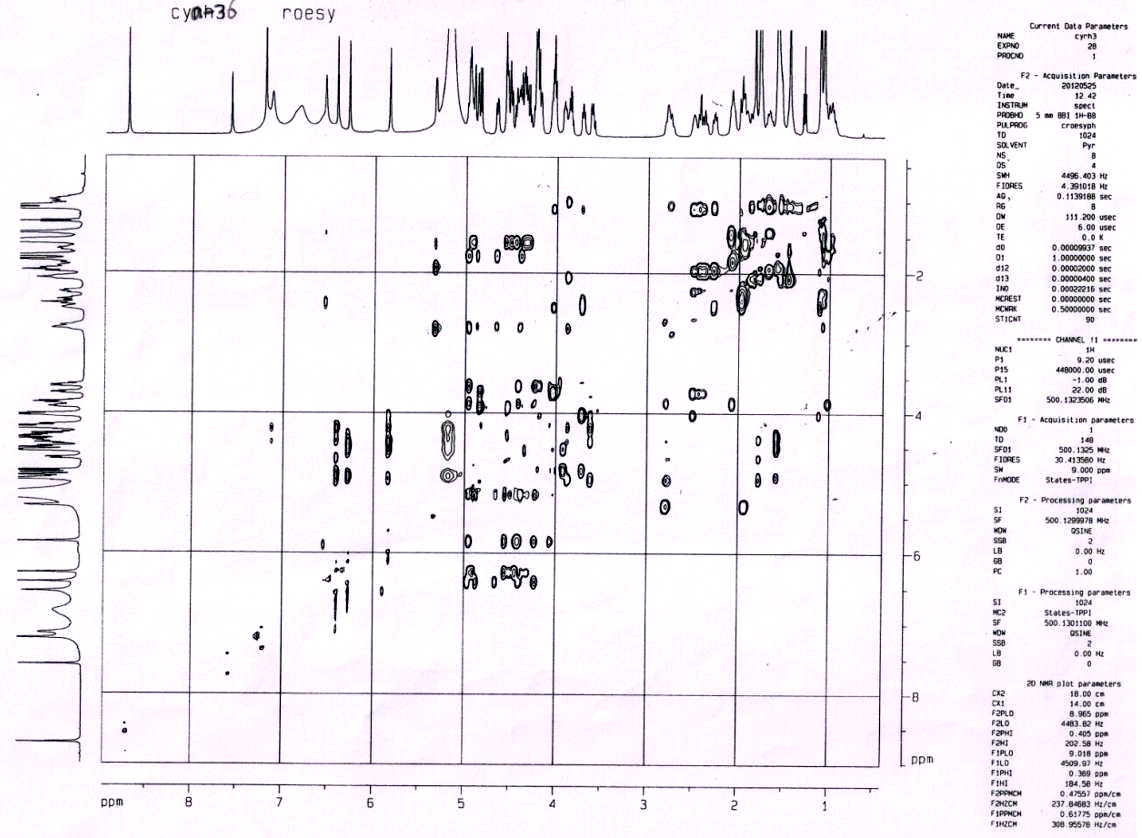


**Figure 6S.** ROESY spectrum of compound **1** (pyridine-*d*_5_, 500 MHz).


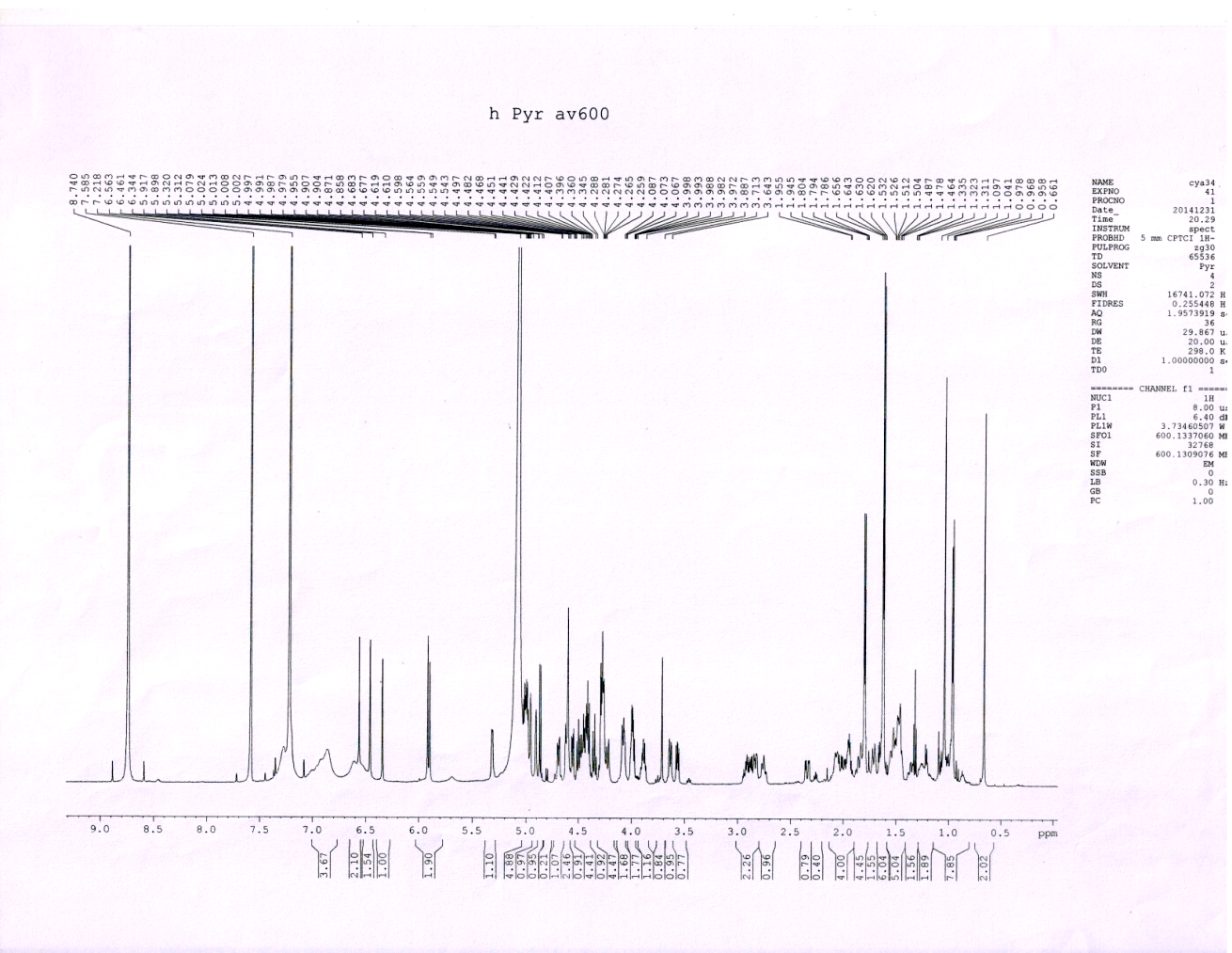


**Figure 7S.** ^1^H NMR spectrum of compound **2** (pyridine-*d*_5_, 600 MHz).


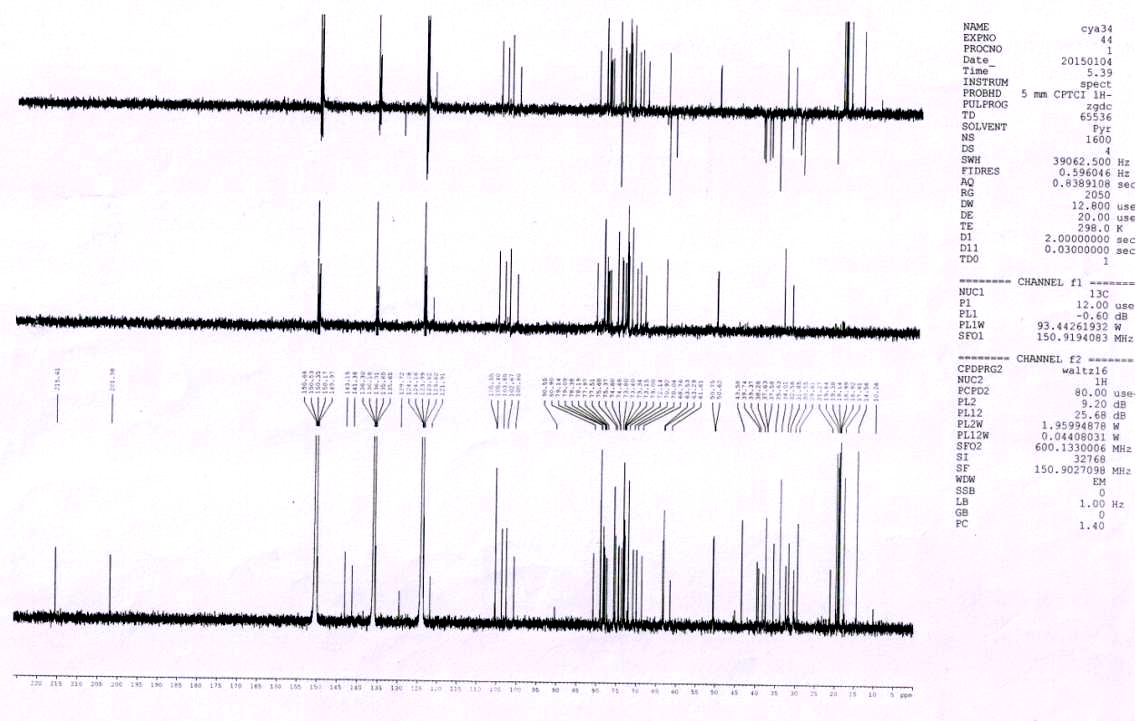


**Figure 8S.** ^13^C NMR spectrum of compound **2** (pyridine-*d*_5_, 150 MHz).


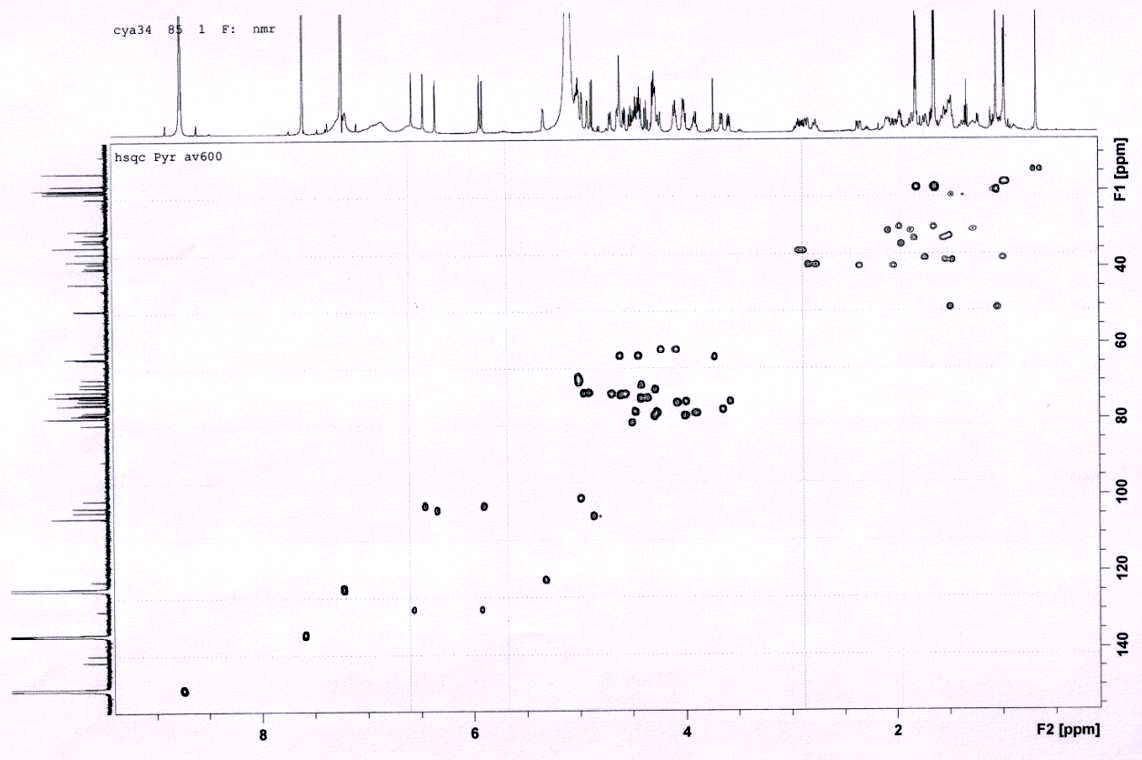


**Figure 9S.** HSQC spectrum of compound **2** (pyridine-*d*_5_, 600 MHz).


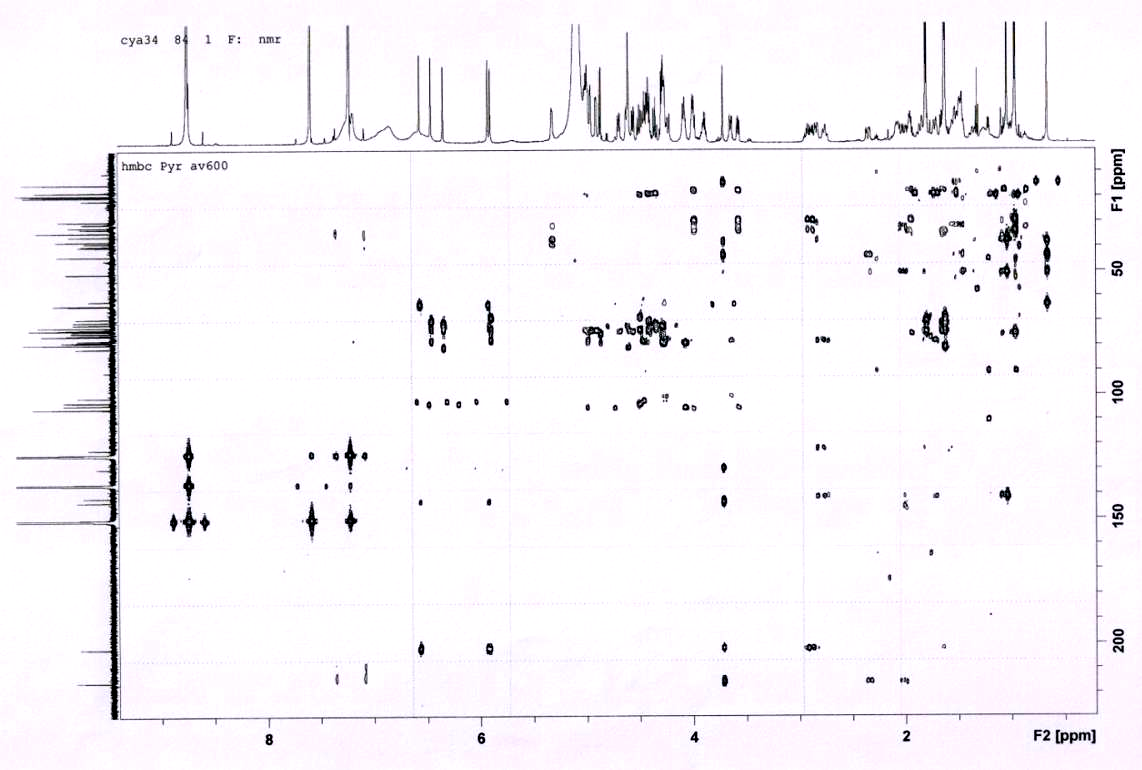


**Figure 10S.** HMBC spectrum of compound **2** (pyridine-*d*_5_, 600 MHz).


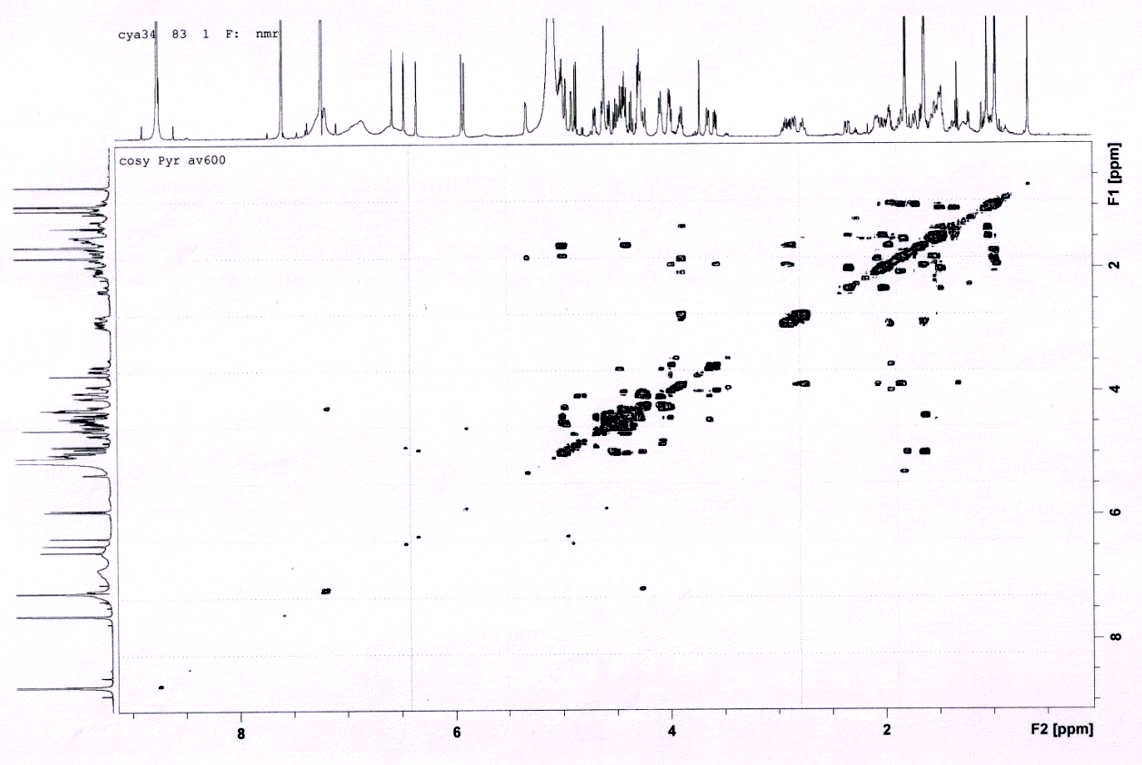


**Figure 11S.** ^1^H-^1^H COSY spectrum of compound **2** (pyridine-*d*_5_, 600 MHz).


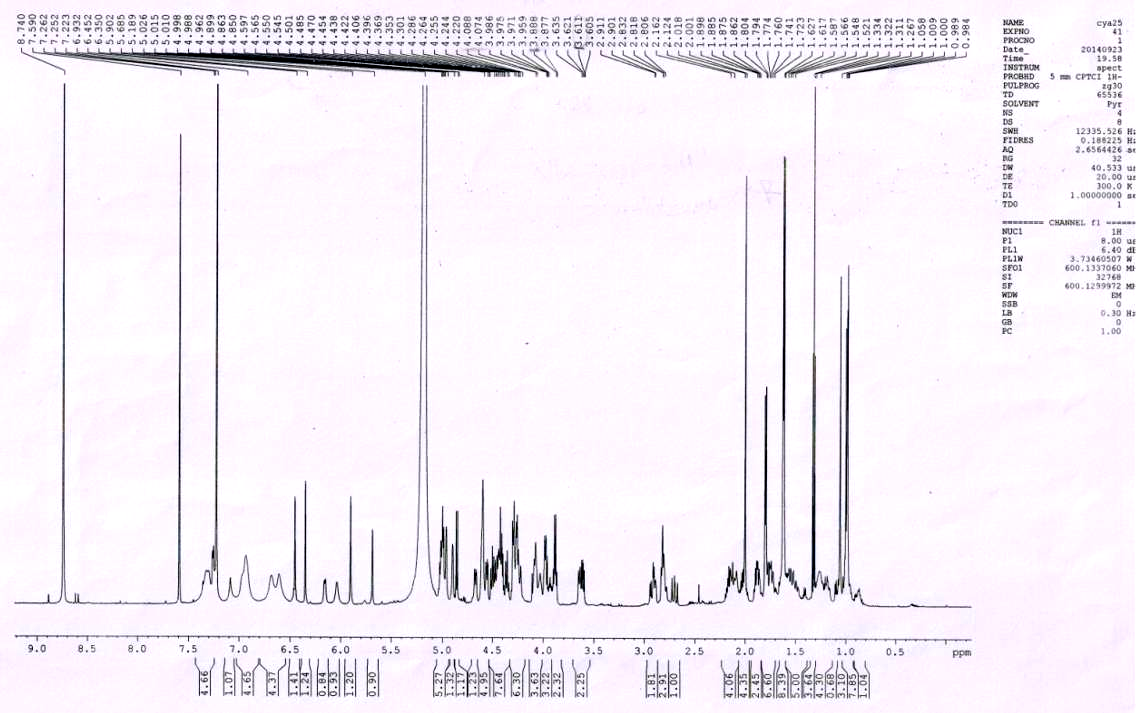


**Figure 12S.** ^1^H NMR spectrum of compound **3** (pyridine-*d*_5_, 600 MHz).


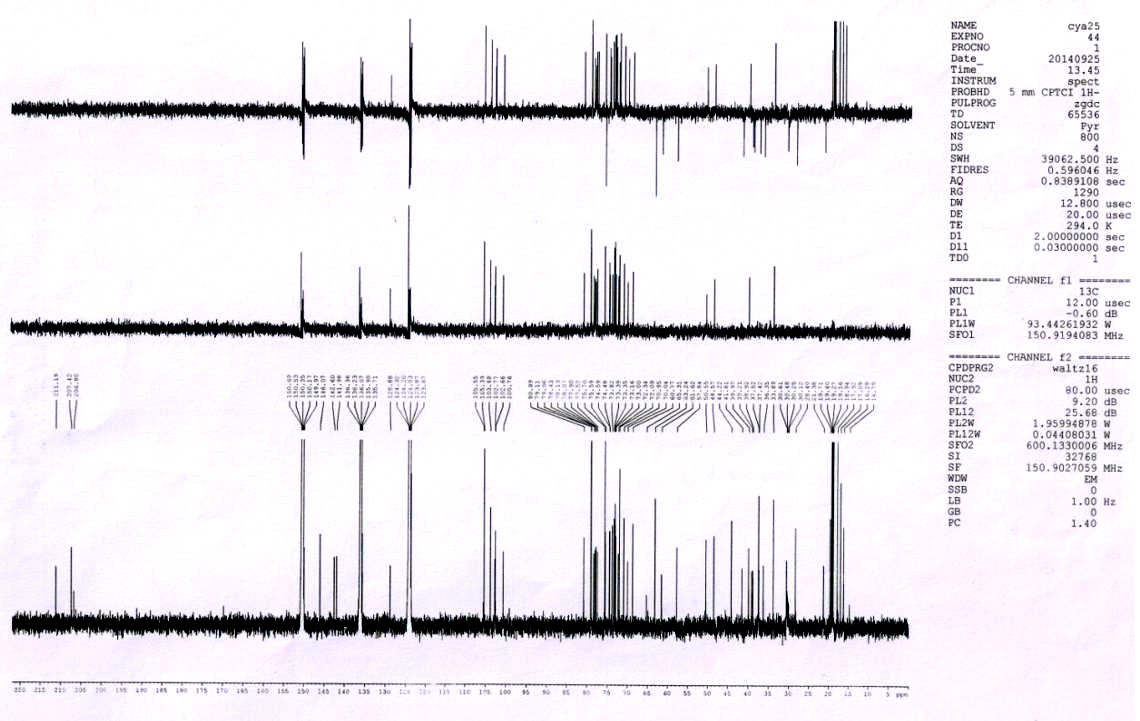


**Figure 13S.** ^13^C NMR spectrum of compound **3** (pyridine-*d*_5_, 150 MHz).


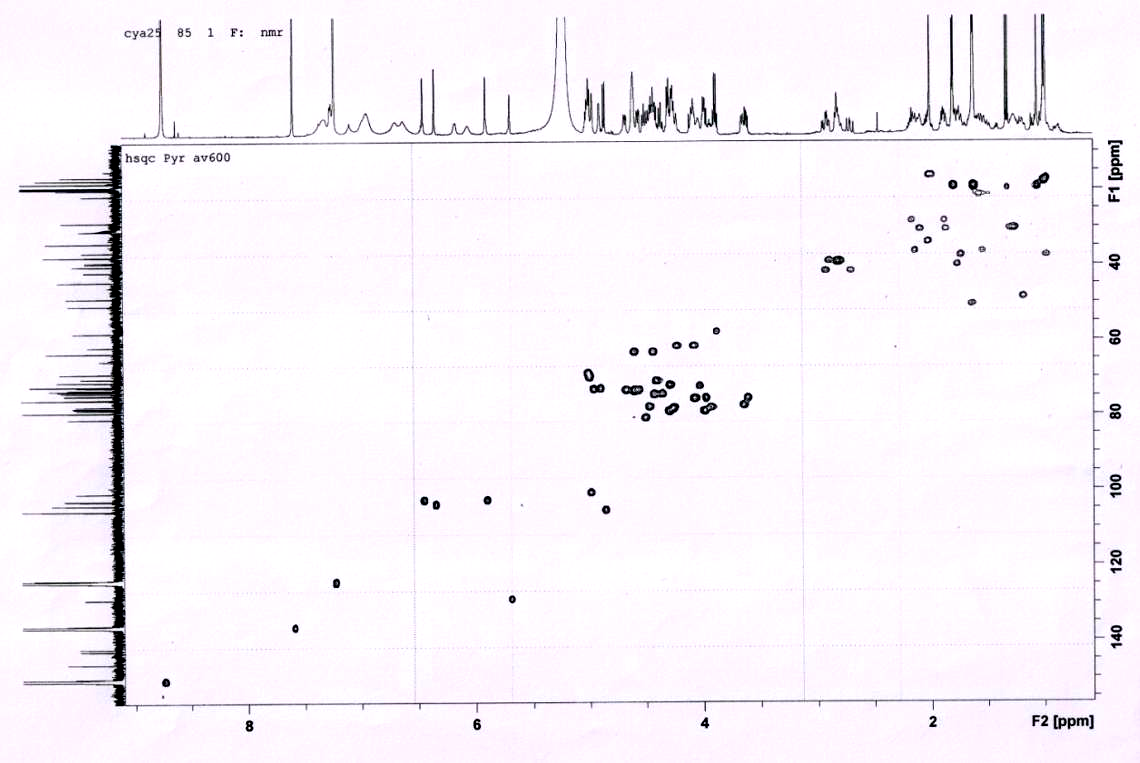


**Figure 14S.** HSQC spectrum of compound **3** (pyridine-*d*_5_, 600 MHz).


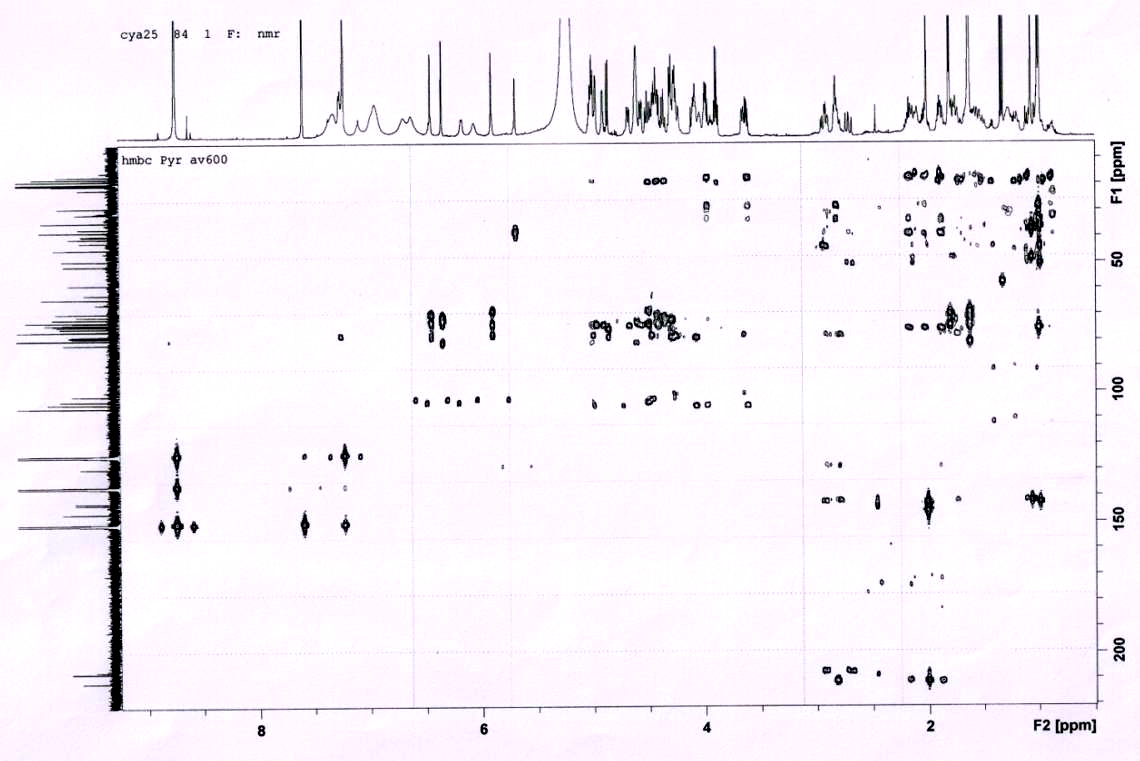


**Figure 15S.** HMBC spectrum of compound **3** (pyridine-*d*_5_, 600 MHz).


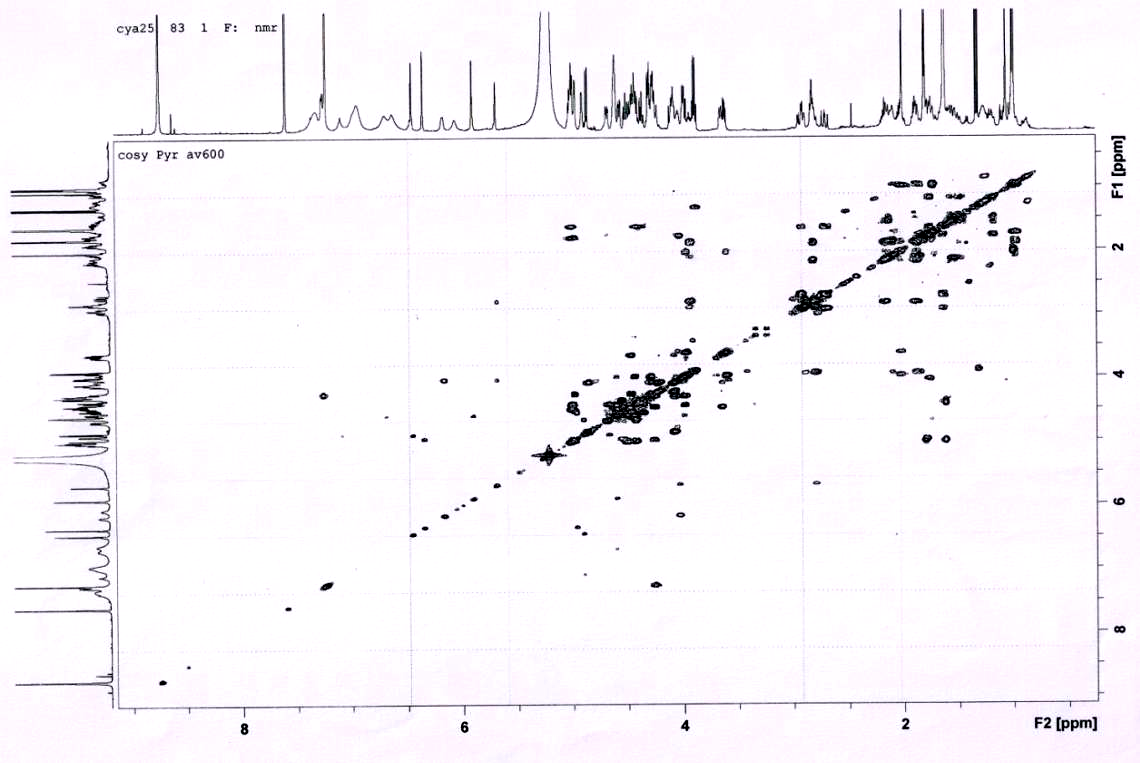


**Figure 16S.** ^1^H-^1^H COSY spectrum of compound **3** (pyridine-*d*_5_, 600 MHz).


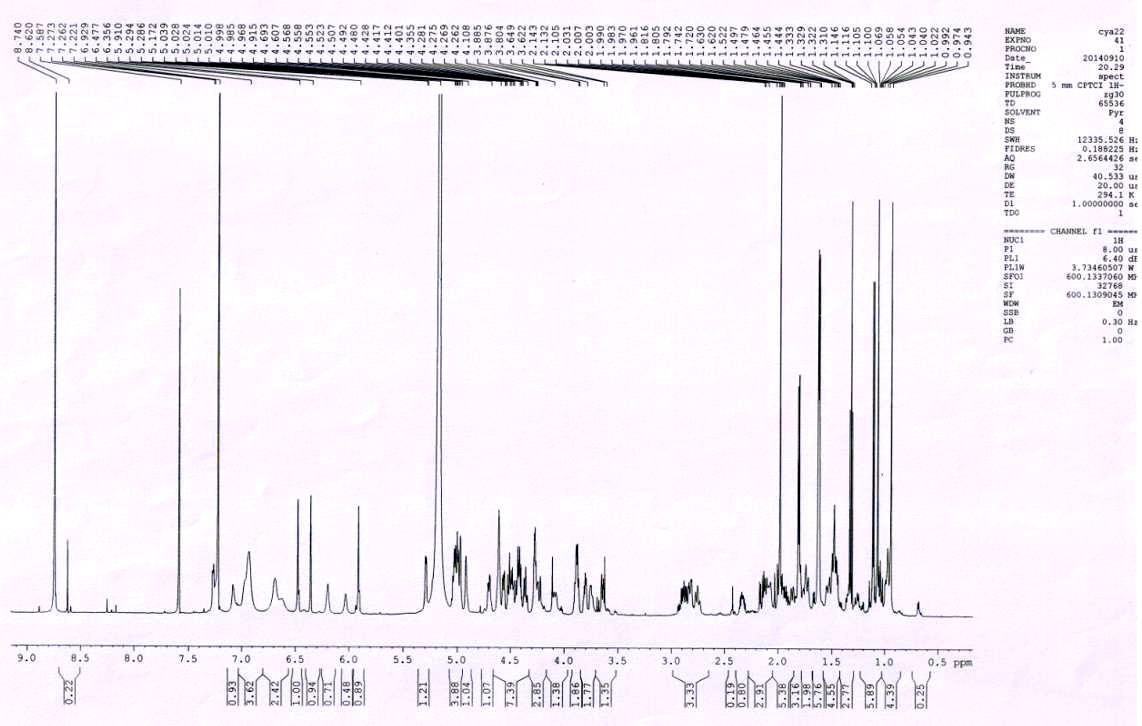


**Figure 17S.** ^1^H NMR spectrum of compound **4** (pyridine-*d*_5_, 600 MHz).


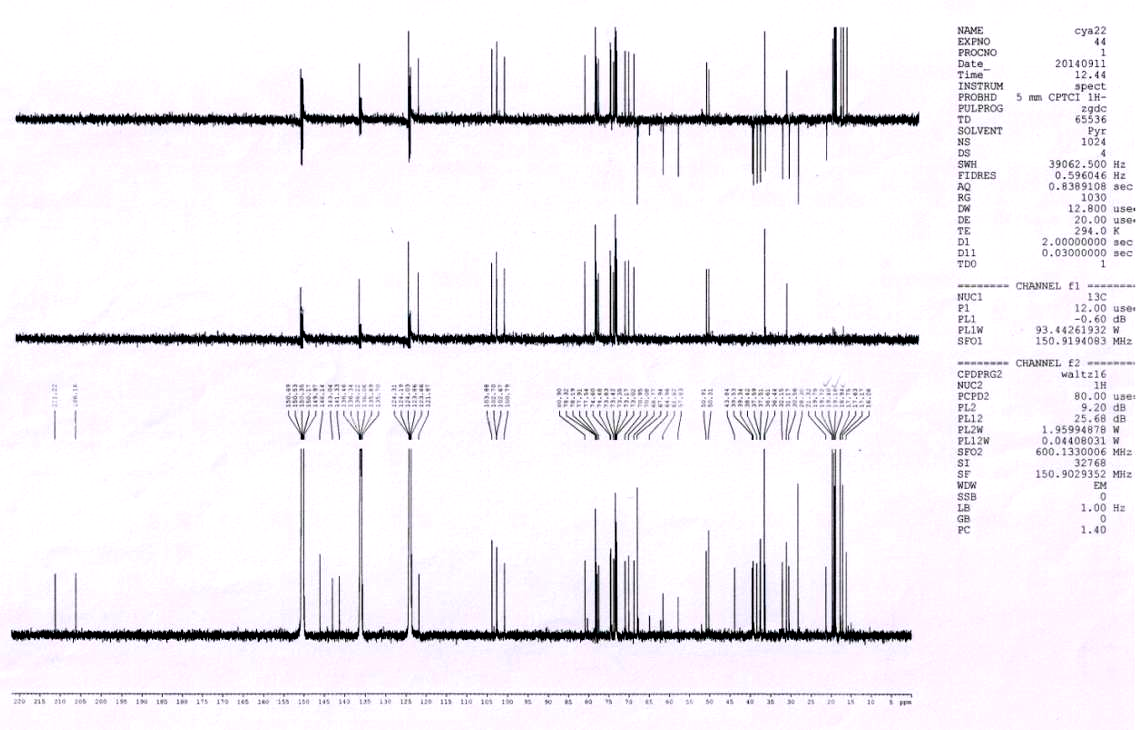


**Figure 18S.** ^13^C NMR spectrum of compound **4** (pyridine-*d*_5_, 150 MHz).


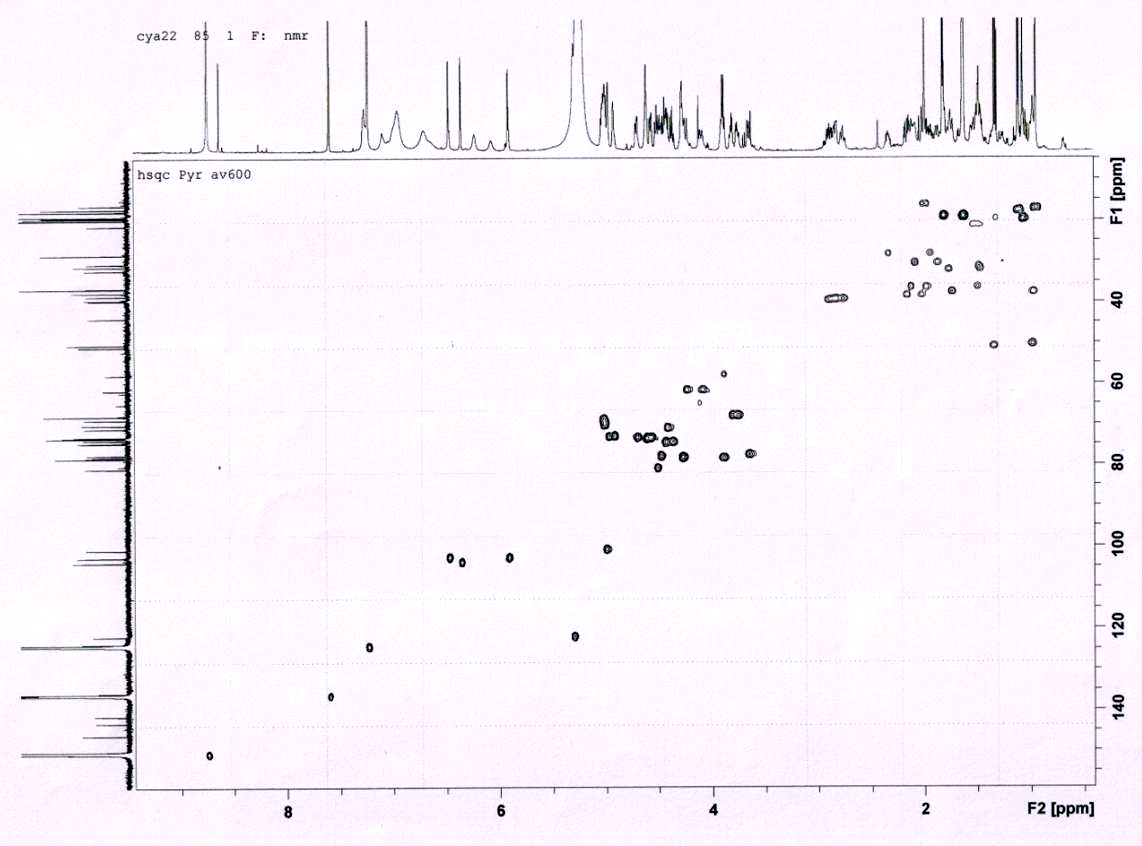


**Figure 19S.** HSQC spectrum of compound **4** (pyridine-*d*_5_, 600 MHz).


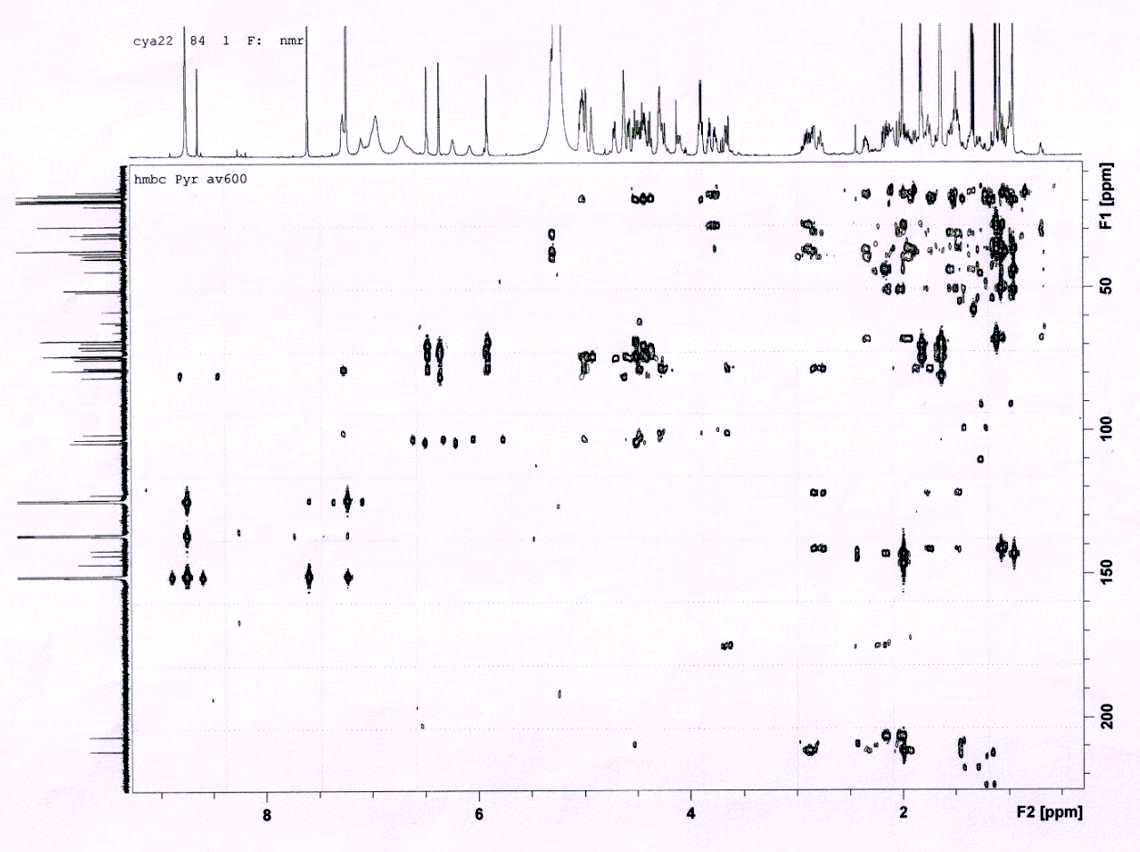


**Figure 20S.** HMBC spectrum of compound **4** (pyridine-*d*_5_, 600 MHz).


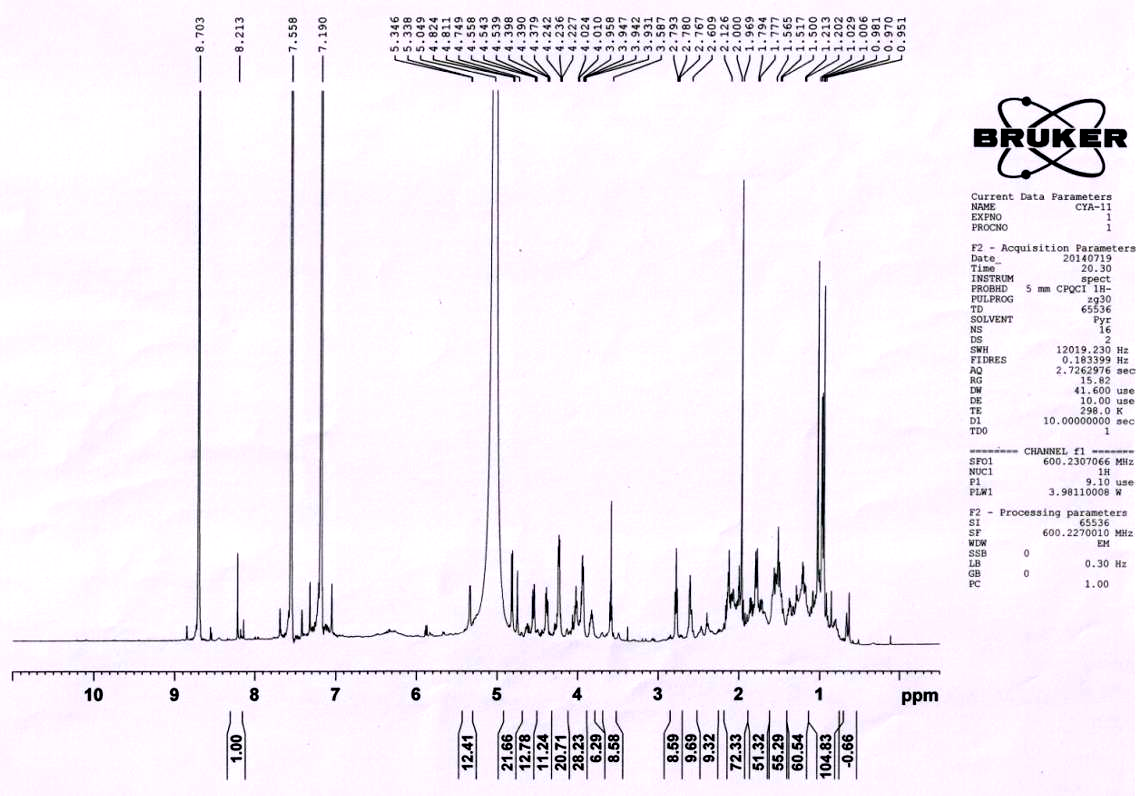


**Figure 21S.** ^1^H NMR spectrum of compound **5** (pyridine-*d*_5_, 600 MHz).


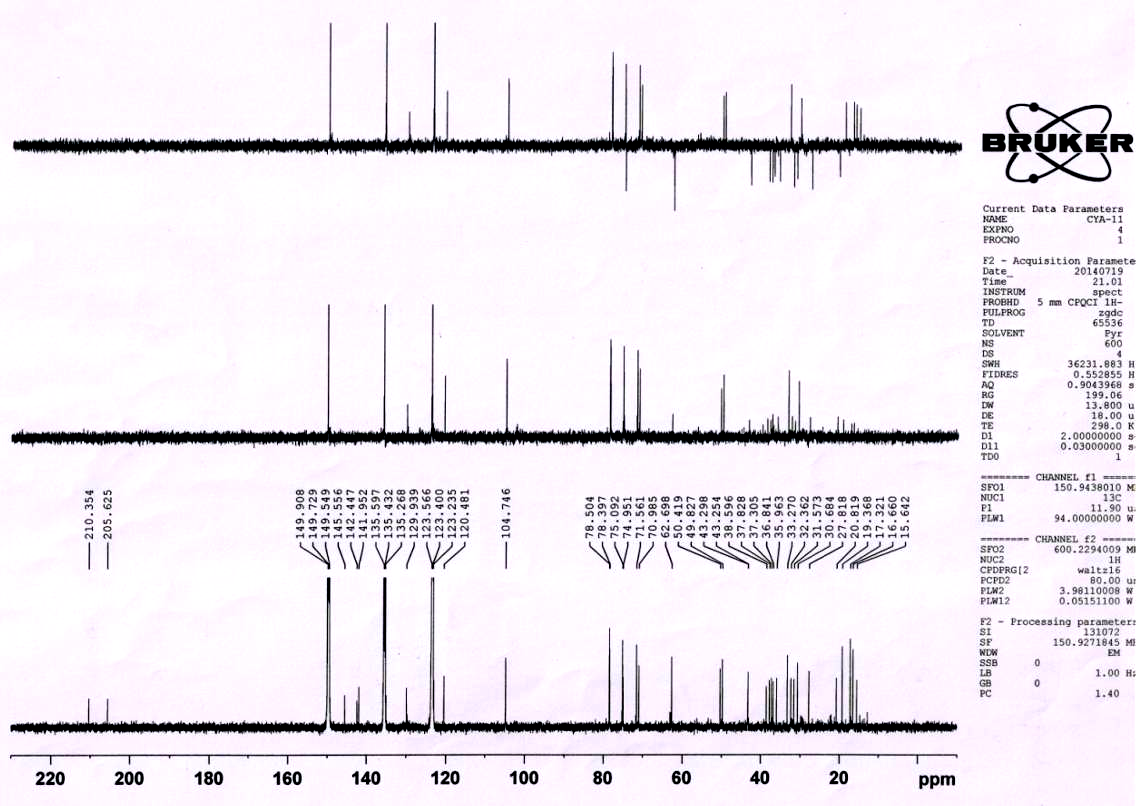


**Figure 22S.** ^13^C NMR spectrum of compound **5** (pyridine-*d*_5_, 150 MHz).


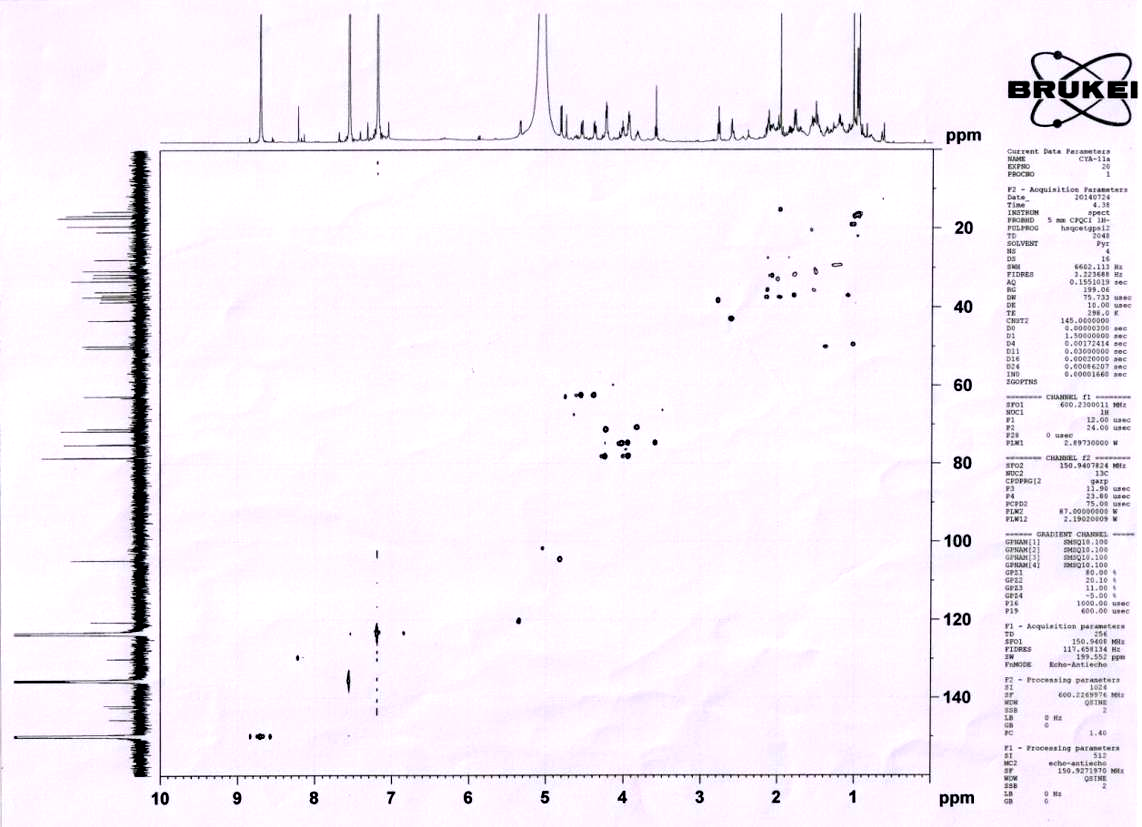


**Figure 23S.** HSQC spectrum of compound **5** (pyridine-*d*_5_, 600 MHz).


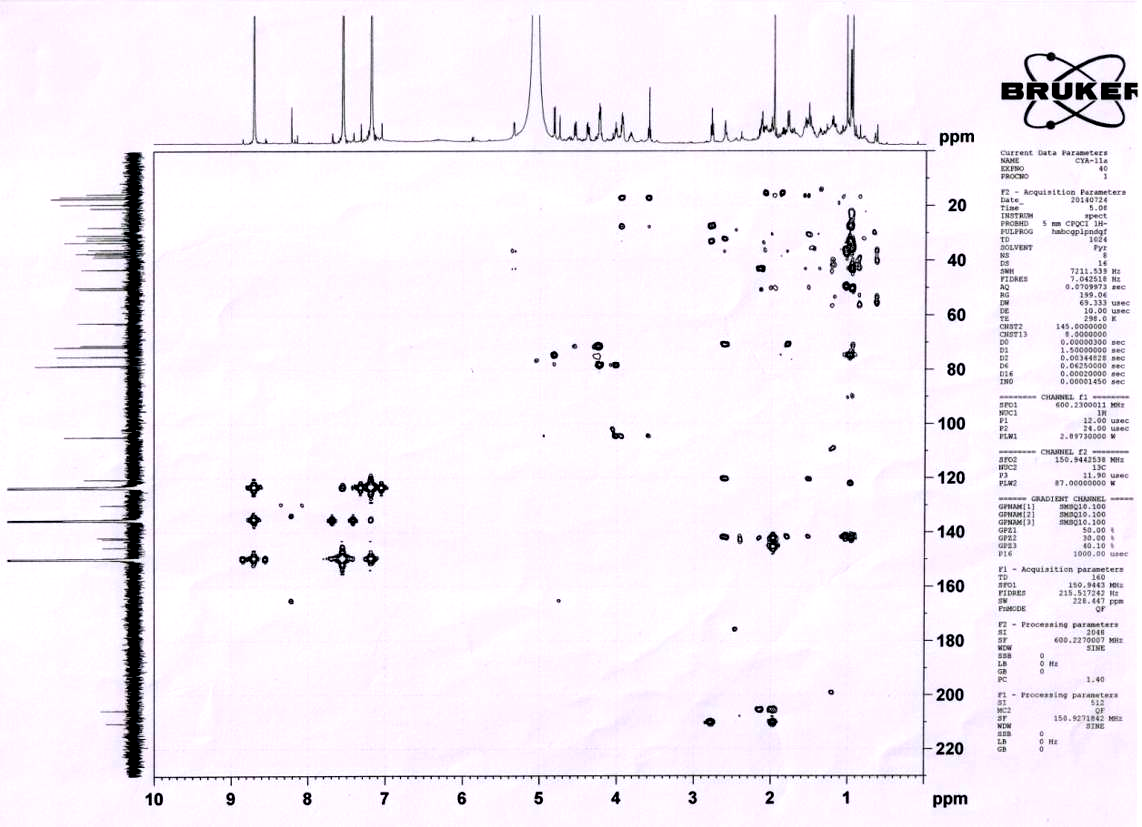


**Figure 24S.** HMBC spectrum of compound **5** (pyridine-*d*_5_, 600 MHz).
